# Triterpenes from the Mushroom *Hypholoma lateritium*: Isolation, Structure Determination and Investigation in Bdelloid Rotifer Assays

**DOI:** 10.3390/molecules24020301

**Published:** 2019-01-15

**Authors:** Bayar Chuluunbaatar, Zoltán Béni, Miklós Dékány, Bernadett Kovács, András Sárközy, Zsolt Datki, Lilla Mácsai, János Kálmán, Judit Hohmann, Attila Ványolós

**Affiliations:** 1 Department of Pharmacognosy, University of Szeged, Eötvös u. 6, H-6720 Szeged, Hungary; ch_bayaraa@pharmacognosy.hu(B.C.); kovacs.bernadett@pharmacognosy.hu (B.K.); sarkozy@pharmacognosy.hu (A.S.); hohmann@pharm.u-szeged.hu (J.H.); 2 Spectroscopic Research, Gedeon Richter Plc., Gyömrői út 19-21, H-1103 Budapest, Hungary; z.beni@richter.hu (Z.B.); M.Dekany@richter.hu (M.D.); 3 Department of Psychiatry, Faculty of Medicine, University of Szeged, Kálvária sgt. 57, H-6725 Szeged, Hungary; datkizsolt@gmail.com (Z.D.); macsai.lilla@gmail.com (L.M.); kalman.janos@med.u-szeged.hu (J.K.); 4 Interdisciplinary Centre for Natural Products, University of Szeged, Eötvös u. 6, H-6720 Szeged, Hungary

**Keywords:** *Hypholoma lateritium*, mushroom, triterpenes, toxicity, bdelloid rotifer

## Abstract

Twelve compounds (**1**–**12**) were isolated from the methanol extract of brick cap mushroom (*Hypholoma lateritium* (Schaeff.) P. Kumm.). The structures of the compounds were elucidated using extensive spectroscopic analyses, including NMR and MS measurements. Lanosta-7,9(11)-diene-12β,21α-epoxy-2α,3β,24β,25-tetraol (**1**) and 8-hydroxy-13-oxo-9*E*,11*E*-octa-decadienoic acid (**2**) were identified as new natural products, together with ten known compounds, from which 3β-hydroxyergosta-7,22-diene (**4**), demethylincisterol A2 (**5**), cerevisterol (**6**), 3β-*O*-glucopyranosyl-5,8-epidioxyergosta-6,22-diene (**7**), fasciculol E (**9**), and uridine (**12**) were identified in this species for the first time. The isolated triterpenes (**1**, **3**–**11**) were investigated for their toxicity in vivo using bdelloid rotifer assays. Most of the examined steroids in general showed low toxicity, although the effects of the compounds varied in a wider range from the non-toxic lanosta-7,9(11)-diene-12β,21α-epoxy-2α,3β,24β,25-tetraol (**1**) to the significantly toxic cerevisterol (**6**), with substantial dependence in some cases on the presence of nutrient in the experimental environment.

## 1. Introduction

Over human history, mushrooms have acquired a good reputation as a popular and valuable foodstuff, being low in calories but high in essential amino acids, vitamins, and fiber. They can be integrated into a versatile and balanced diet, representing not only a tasty and healthy food, but also a rich source of biologically active natural products with therapeutic potential. According to the report of the Food and Agriculture Organization (FAO), there are records of more than 1100 mushrooms with varying degrees of edibility from 85 countries around the world [1]. The question of edibility is fairly subjective; the comestibility of some mushrooms can vary from one region to another, while certain species are eaten only after they are processed in a specific way. 

A particular case is represented by *Hypholoma lateritium* ((Schaeff.) P. Kumm. (syn. *Hypholoma sublateritium* (Fr.) Quél. and *Naematoloma sublateritium* (Fr.) P. Karst.)), also known as brick cap, which is a popular edible mushroom in Japan, Korea, and the United States, but in Europe it is considered inedible or even poisonous [2,3]. *H. lateritium* is a wood-decay fungus growing in clusters on hardwood logs and stumps, widely distributed throughout Europe, North America, and the Far East. Compared to its more common relative, the poisonous sulfur tuft (*Hypholoma fasciculare*), the chemistry and pharmacology of *H. lateritium* are less known. This species produces several triterpenes, e.g., fasciculols B and C, their depsipeptides (fasciculols D and F), and sublateriols A–C [4,5]. Naematolin, a caryophyllane derivative with cytotoxic activity, was isolated from cultures of *H. lateritium* [6]. Attempts have been made to characterize the possible mechanism of action behind the antitumor and anti-inflammatory effects of *H. lateritium* extracts [7,8], as well as to explore the antimicrobial, antioxidant, and xanthine oxidase inhibitory properties of this species [9,10,11]. The current study was performed to identify the major secondary metabolites of *H. lateritium* and characterize their toxicity in bdelloid rotifer assays. Bdelloid rotifers, as micro-invertebrates, are widely used animal models in toxicity-, aging-, and longevity-related research [12,13,14]. These organisms are multicellular animals with well-defined anatomical characteristics, possessing a ciliated head structure, bilateral ovaries, mastax, ganglia, muscle, digestive, nervous, and secretory systems, and photosensitive and tactile organs [15,16]. Taking into account the above-mentioned characteristics together with their short lifespan and specific measurable phenotypic features and viability markers [17], bdelloids are useful as in vivo toxicological and lifespan models.

## 2. Results and Discussion

**Figure 1:**
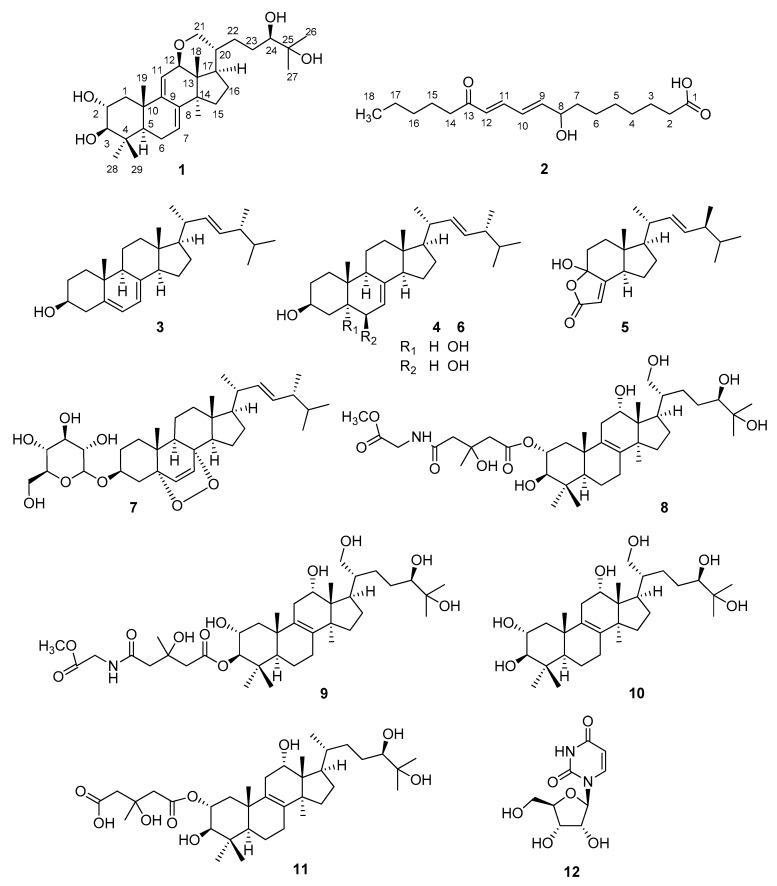
Structures of compounds isolated from *H. lateritium.*

As part of our ongoing effort to search for biologically-active natural fungal products, our attention has been drawn to the mushroom *H. lateritium*, a species known for its controversial edibility, but investigated to a weaker extent. In this vein, our main goal was to identify the characteristic compounds of *H. lateritium* and to evaluate their toxicity in vivo using bdelloid rotifer assays. The collected fruiting bodies of *H. lateritium* were freeze-dried, and then were extracted with methanol on room temperature. The crude extract was subjected to solvent-solvent partition with *n*-hexane, chloroform, and then ethyl acetate. The obtained *n*-hexane, chloroform, and ethyl acetate phases were applied to an extensive separation process, using a combination of flash chromatography steps on normal and reversed phases to afford 12 compounds (Figure 1). 

**Figure 2:**
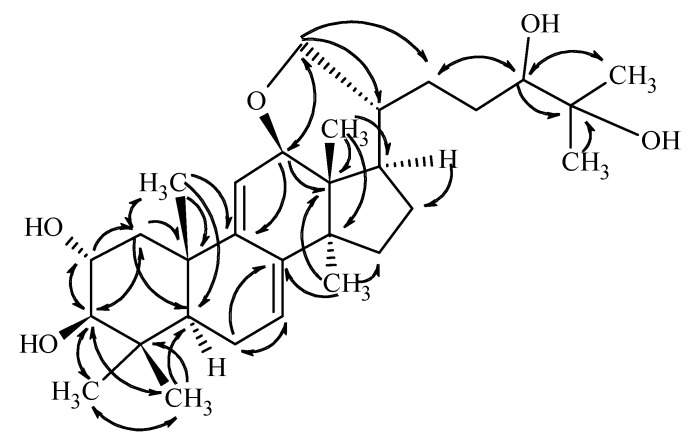
Key HMBC correlations of compound **1.**

Compound **1** was isolated as a colorless gum. Based on the HRESIMS and ^13^C-NMR data, its molecular formula was determined to be C_30_H_48_O_5_. Consecutive analysis of the ^1^H, ^13^C, COSY, HSQC, and HMBC NMR spectra (see Supplementary Materials) suggested the presence of seven tertiary methyl group, two olephinic, four oxygenated, and three aliphatic methines, an oxygenated and six aliphatic methylenes, together with five quaternary carbons. The determined data were quite similar to those reported for sublateriol C [5], except for the ^1^H and ^13^C resonances assigned to ring D, and C-12, C-18, C-20, C-21, and C-28. The observed HMBC correlations between H-12 and C-21 and between H-21 and C-12 (Figure 2) suggested that instead of two hydroxyl groups present in sublateriol C, an epoxy group between C12 and C21 is present in **1**. This ring closure is in accordance with the H_2_O difference obtained between the elementary compositions of sublateriol C and **1**, and explains the chemical shift differences obtained for the protons and carbons close to C-12 and C-20 centers.

**Figure 3:**
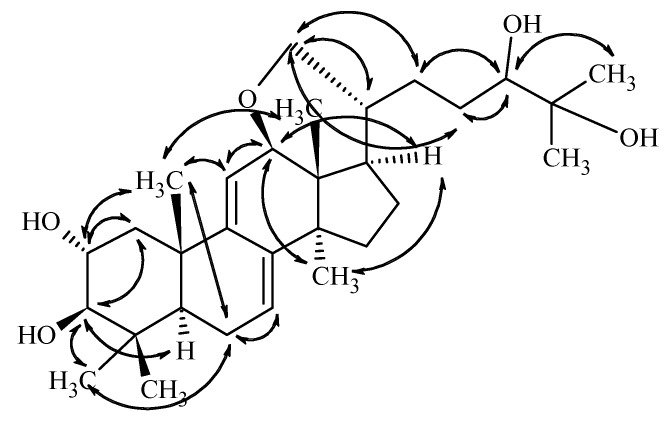
Key NOE correlations determined from the ROESY spectrum of compound **1.**

Based on the key NOE correlations (Figure 3) observed between H-2/H-19, H-3/H-5/H-30, H-12/H-28/H-17, and H-18/H-20, the above presented stereochemistry is suggested. 

The configuration of C-24 center, however, could not be assigned on this basis, and the OH group is only tentatively given as beta-positioned. This suggestion is firstly based on the assumption that the ring closure does not drastically change the conformation of the side chain. In this case, the observed doublet nature of H-24 with coupling constants of 9.8 and 1.6 Hz, which are closely similar to those reported for sublateriol A [5] or other isolated fasciculic acid and fasciculol derivatives, having the same side chain [18,19], suggest a similar configuration of the C-24 center. Secondly, making the assumption that similar metabolic pathways lead to sublateriol C and **1** in the same mushroom species, a similar β orientation of 24-OH is suggested. Putting all these together, the structure of compound **1** is suggested as lanosta-7,9(11)-diene-12β,21α-epoxy-2α,3β,24β,25-tetraol.

**Figure 4:**
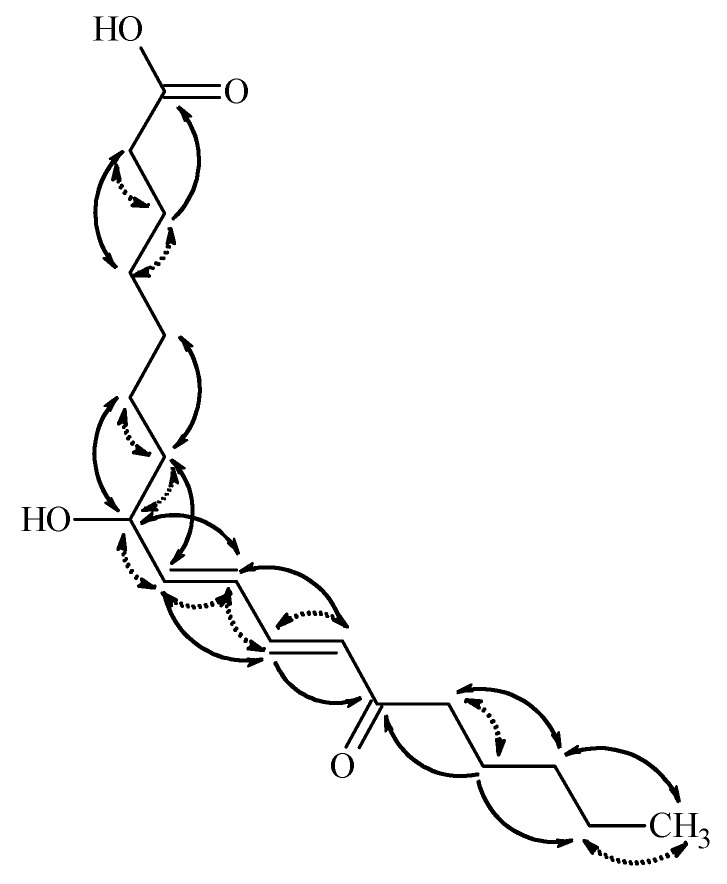
Key HMBC (solid line) and COSY (dotted line) correlations in compound **2.**

Compound **2** was obtained as colorless gum. Its molecular formula, C_18_H_29_O_4_, was established from HR-ESI MS measurement giving a pseudomolecular ion peak at *m*/*z* 309.20673 ([M − H]^−^) in the negative ion mode. In accordance with this elementary composition, the ^13^C-NMR spectrum presented eighteen carbon resonances. Based on the ^1^H and edited HSQC spectra one methyl, ten methylenes, five methines, and two quaternary carbons were present in the isolated compound. The ^13^C chemical shifts of the quaternary carbons (182.9 and 204.0 ppm) suggested the presence of a carboxylic acid and a ketone functionality. The multiplicities and coupling constants of the ^1^H resonances belonging to the methine protons suggested the presence of two conjugated double bonds, both in *E* configuration, connected to a hydroxylated methine and to a keto group. Putting this information together led to the conclusion that the isolated compound is a hydroxyl-oxo-octadecadienoic acid derivative. The positions of the functional groups in the fatty acid chain were unambiguously evidenced on the basis of the COSY and HMBC correlations presented in Figure 4. 

Thus, the HMBC correlations observed between H-14 and C-16, C-12, C-13, and C-15, and between H-18 and C-16 and C-17, suggested that the keto group is at position 13, while the OH is connected to C-8. The HMBC correlation of H-11 and H-12 to C-14, and those of H-9 and H-10 to C-8 confirmed these suggestions. Based on these data, the 8-hydroxy-13-oxo-9*E*,11*E*-octadecadienoic acid structure is suggested for **2**. The absolute stereochemistry of C8 center was not determined. Ergosterol (**3**), 3β-hydroxyergosta-7,22-diene (**4**), cerevisterol (**6**), and 3β-*O*-glucopyranosyl-5,8-epidioxyergosta-6,22-diene (**7**) were identified by comparing their chromatographic and spectroscopic data with those of authentic samples. Compounds **5** and **8**–**12** were structurally characterized on the basis of NMR and MS spectroscopic data (see Supplementary Materials) and confirmed by comparing them to those reported earlier in the literature [4,5,18,19,20,21,22,23,24]. Demethylincisterol A2 (**5**) is a highly degraded sterol isolated first from a marine sponge of *Homaxinella* sp. [24]. Fasciculol E (**9**) was first identified in the sporocarps of *Hypholoma fasciculare* [19], while fasciculol F (**8**), fasciculol C (**10**), and fasciculic acid B (**11**) were previously isolated from *H. lateritium* [4]; compound **12** was determined as uridine.

**Figure 5:**
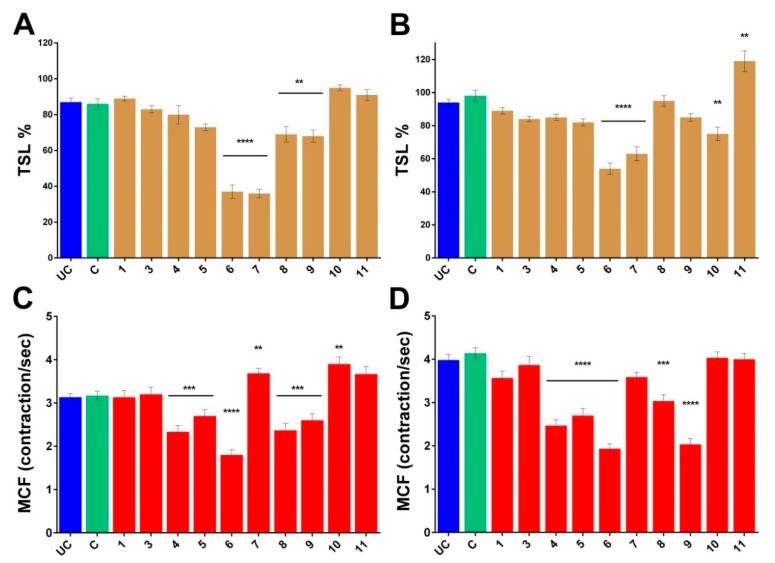
Normalized rotifer characteristics. Changes in the TSL values of the *Philodina acuticornis* after 3-day treatment (**A**) without feeding and (**B**) with feeding, compared to the group C. Changes in the MCF values of the *Philodina acuticornis* after 3-day treatment (**C**) without feeding and (**D**) with feeding, compared to the group C. UC: untreated control, C: control with 0, 1% DMSO. **1**, **3**–**11**: compounds. TSL: toxicity and survival lifespan (*n* = 12, well). MCF: mastax contraction frequency (*n* = 30, individual rotifer). Values are the mean ± SEM; *p* ** ≤ 0.01, *p* *** ≤ 0.001 and *p* **** ≤ 0.0001.

The isolated compounds were subjected to bdelloid rotifer assay in order to gain information about their toxicity and biological activity. Two viability markers of *Philodina acuticornis* have been used to measure the effect of compounds **1** and **3**–**11** in terms of survival and resilience. Decreases or increases in the toxicity and survival lifespan (TSL) and in the mastax contraction frequency (MCF) are in correlation with the physiological state of individuals. The changes in TSL values, which provides mortality rate, are rather straightforward results. The MCF index assays the chewing organ function, providing information about the effect of the compounds on organs level and gives a more complex image to the results. Compounds which caused significant decrease in the number of survivors were cerevisterol (**6**) and 3β-*O*-glucopyranosyl-5,8-epidioxyergosta-6,22-diene (**7**) (see Figure 5).

Significant decrease was observed in the MCF values of 3β-hydroxyergosta-7,22-diene (**4**), demethylincisterol A2 (**5**), cerevisterol (**6**), fasciculol E (**9**), and fasciculol F (**8**). Cerevisterol (**6**) and 3β-*O*-glucopyranosyl-5,8-epidioxyergosta-6,22-diene (**7**) were significantly toxic, however the latter in the presence of nutrients proved to be less toxic, and the MCF values significantly increased in the survivors. The complete opposite was observed with fasciculol C (**10**), which demonstrated toxic effect with feeding, but increased the MCF values without nutrients. Fasciculol E (**9**) caused an overall significant decrease in the viability values, with strong dependence on the presence or lack of nutrients. Among the examined compounds, cerevisterol (**6**) proved to be the most toxic, while lanosta-7,9(11)-diene-12β,21α-epoxy-2α,3β,24β,25-tetraol (**1**) had no harmful effect at all. Fasciculic acid B (**11**) exhibited unique effects, since it was used by rotifers simply as a food source. Overall, we can state that most of the investigated steroids in general had low toxicity, although the effect of compounds varied in a wider range from non-toxic lanosta-7,9(11)-diene-12β,21α-epoxy-2α,3β,24β,25-tetraol (**1**) to significantly toxic cerevisterol (**6**), with strong dependence in some cases on the presence of nutrients in the experimental environment.

## 3. Materials and Methods

Optical rotations were measured with a Perkin-Elmer 341 polarimeter. Flash chromatography was carried out on a CombiFlash®Rf+Lumen Instrument (Teledyne ISCO, Lincoln, NE, USA) with integrated UV, UV-VIS, and ELS detection using RediSep Rf Gold normal and reversed phase flash columns (4, 12, 40 and 80 g) (Teledyne Isco, Lincoln, NE, USA). HRMS analyses were performed on a LTQ FT Ultra (Thermo Fisher Scientific, Bremen, Germany) system. The samples were dissolved in methanol. ESI ionization was used in all cases operating in positive or in case of compound **7** in the negative ion mode. HR-MS-MS were acquired using CID fragmentation method applied on the quasimolecular ion peaks (protonated/deprotonated molecular ion peaks or the sodium adduct ions or the molecular ion peaks with water losses). Data acquisition and analysis were accomplished with Xcalibur software version 2.0 (Thermo Fisher Scientific). NMR data were acquired on a Varian 800, Bruker Avance II HD 500 (Bruker, Billerica, MA, USA) or a Bruker AVANCE II HD 400 MHz spectrometer equipped with a ^13^C enhanced salt tolerant cryoprobe, a TCI cold probe, or liquid nitrogen cooled Prodigy probe. MeOD-*d_4_* was used as solvent in all cases. Chemical shifts are reported in the delta scale relative to the residual solvent signals (3.31 and 49.15 ppm for ^1^H and ^13^C, respectively). Standard one and two dimensional NMR spectra were recorded in all cases, using the pulse sequences available in the VNMRJ 3.2 (Agilent Technologies, Santa Clara, CA, USA) or in TopSpin 3.5 sequence libraries (Bruker). Data analysis and interpretation were performed within ACD/Labs 2017.1.3 NMR Workbook Suite.

### 3.1. Mushroom Material

Fruiting bodies of *Hypholoma lateritium* were collected in September 2015 from the environs of Bakonybél, Hungary, and identified by Attila Sándor (Hungarian Mycological Society). A voucher specimen (collection number H018) has been deposited at the Department of Pharmacognosy, University of Szeged, Szeged, Hungary.

### 3.2. Extraction and Isolation

The mushroom material (6.5 kg) was freeze dried and then the dry sample (630 g) was extracted with methanol (7 L) at room temperature. After concentration, the methanol extract (92 g) was dissolved in 50% aqueous MeOH and subjected to solvent-solvent partition using *n*-hexane (5 × 500 mL), chloroform (5 × 500 mL), and then ethyl acetate (5 × 500 mL). The *n*-hexane soluble phase (25 g) was applied to flash chromatography (FC) on silica gel using gradient system of *n*-hexane–acetone (0% to 100%, t = 60 min), affording 7 combined fractions (H 1–7). Fractions H 1–2 (2.4 g) were further separated by FC applying an *n*-hexane–acetone solvent system (0% to 30%, t = 45 min), which resulted in compounds **3** (0.92 g) and **4** (0.63 g). Fraction H 3 was first separated by FC on silica gel using *n*-hexane–acetone eluent with increasing polarity (0% to 40% acetone, t = 50 min), then was further purified on reversed phase using water–acetonitrile solvent system (30% to 60% acetonitrile, t = 50 min), and led to the isolation of **6** (12.4 mg). Fraction H 4 (0.49 g) was subjected to normal phase FC using an *n*-hexane-acetone system (0% to 40% acetone, t = 50 min) to give compound **5** (3.6 mg). The combined fraction H 7 (0.83 g) was analyzed in similar conditions (*n*-hexane–acetone, 10% to 50% acetone, t = 55 min), resulting in compound **7** (3.1 mg). The chloroform soluble phase (18 g) was first separated by normal phase FC, applying a solvent system of *n*-hexane–acetone (0% to 100%, t = 60 min) to obtain 11 major combined fractions (C 1–11), while the ethyl acetate phase (12 g) was subjected to normal phase FC using a solvent system of chloroform–methanol, which resulted in six major fractions (E 1–6). Fraction C 2 (0.34 g) was separated on normal phase (solvent system *n*-hexane–acetone, 0% to 40% acetone, t = 50 min), followed by reversed phase flash chromatography purification (30% to 60% acetonitrile, t = 50 min) to give compounds **1** (3.3 mg) and **2** (3.3 mg). Fractions C 7–8 (1.05 g) were further purified on normal (*n*-hexane–acetone, 0% to 40% acetone, t = 45 min) and reversed phase (35% to 60% acetonitrile, t = 50 min) flash chromatography in subsequent steps to afford **8** (0.32 g) and **9** (0.10 g). The combined fractions C 9–11 (3.89 g) and E 1 (1.26 g) were separated by subsequent use of normal (*n*-hexane–acetone, 0% to 40% acetone, t = 50 min) and reversed phase (30% to 70% acetonitrile, t = 50 min) flash chromatography to obtain **10** (0.42 g) and **11** (0.31 g). Finally, compound **12** (28 mg) was isolated from fractions E 5–6 using a chloroform–methanol solvent system (0% to 30% methanol, t = 45 min).

*Lanosta-7,9(11)-diene-12β,21α-epoxy-2α,3β,24β,25-tetraol* (**1**): colorless gum; [α]${}_{D}^{26}$ +7 (MeOH, *c* 0.2), ^1^H-NMR and ^13^C-NMR data, see Table 1. HRESIMS: *m*/*z* 395.33056 [M + H-H_2_O-H_2_O]^+^ (delta = −0.3 ppm; C_28_H_43_O). HR-ESI-MS-MS (CID = 35%; rel. int. %): 377(100); 325(16); 311(51); 307(17); 293(29); 269(12); 251(5).

*8-Hydroxy-13-oxo-9*E*,11*E*-octadecadienoic acid* (**2**): colorless gum; [α]${}_{D}^{26}$ –5 (MeOH, *c* 0.2), ^1^H-NMR and ^13^C-NMR data, see Table 2. HRESIMS: *m*/*z* 309.20673 [M − H]^−^ (delta = −1.3 ppm; C_18_H_29_O_4_). HR-ESI-MS-MS (CID = 55%; rel. int. %): 291(100); 209(8); 195(21); 171(4).

### 3.3. Bdelloid Rotifer Assays

#### 3.3.1. Model Animal

The culturing, harvesting, and monitoring methods of *Philodina acuticornis* (PA; bdelloid rotifer) have been reported in detail in our prior publication [17]. In brief, the animals were cultured in standard medium (SM), a supervised and semi-sterile environment. Clear cultures of PA were kept in standardized cell culturing flasks (cat. no.: 83.3910.302, Sarstedt AG & Co., Nümbrecht, Germany) at 25 °C and under a light/dark cycle of 12:12 h. Rotifers were chosen approximately 5 days after hatching (determined by body size; length 220 ± 10 μm and width 60 ± 5 μm), 1–2 days before the beginning of the reproductive stage. 

#### 3.3.2. Treatment Protocol

As the methodical protocols have been previously reported [17,25], only an overview of the applied techniques is given. After 24 h of the standard isolation process, the rotifers were treated in a 96 well plate (cat. no.: 3695, Costar, Corning Inc., Corning, NY, USA), *n* = 12/well/compounds. Starting rotifer numbers per well: 25 ± 5. For this in vivo experiment, stock solutions were prepared with 1% aqueous DMSO. The stock solutions were added to SM reaching 100 μM final concentrations for the compounds and 0.1% DMSO content. The untreated control group (UC) was grown in SM, while the control group (C) was kept in SM containing 0.1% DMSO (*n* = 12, well, respectively). The status of the specimens under treatment was compared to the group C. This period lasted for 72 h without feeding (toxicity interval), or feeding with homogenized yeast solution (50 μg/mL), which is enough for survival but ceases reproduction. The viability of rotifers was assessed with three different assays, utilizing video recordings with a Nikon D5500 DSLR camera (Nikon Corp., Japan). 

#### 3.3.3. Viability Assays

The impact of the test compounds on the lifespan of rotifers was assessed. The morphological viability markers chosen for evaluation were defined in our previous work [17].

*Toxicity and Survival Lifespan (TSL) Assay.* The TSL index is a life-conditional marker of rotifers’ existence, provides mortality rate (with or without feeding). 

*Mastax Contraction Frequency (MCF) Assay.* The mastax (pharynx) is part of the digestive system. The function of the mastax is to shred the food by periodic opening and closing. To evaluate the viability of rotifers in our experiments, we used the MCF (contraction/sec) as a quantitative viability marker.

#### 3.3.4. Statistics

Data are presented as means ± SEM. Statistical evaluation was performed with GraphPad Prism 7.0b, using One-way ANOVA with post hoc Bonferroni test. Different levels of significance are indicated as follows: *p* ** ≤ 0.01, *p* *** ≤ 0.001 and *p* **** ≤ 0.0001.

#### 3.3.5. Ethical Approval

Our experiments were performed on micro-invertebrates; therefore, according to the current ethical regulations, no specific ethical permission was needed. The investigations were carried out in accordance with globally accepted norms: Animals (Scientific Procedures) Act, 1986, associated guidelines, EU Directive 2010/63/EU for animal experiments, and the National Institutes of Health guide for the care and use of Laboratory Animals (NIH Publications No. 8023, revised 1978). Our animal studies comply with the ARRIVE guidelines. 

## 4. Conclusions

Our current study provides the most comprehensive chemical analysis of the mushroom *H. lateritium*, affording not only novel information on the characteristic secondary metabolites of this species, but also valuable results of in vivo toxicity assays of isolated compounds, which can present an essential basis for future pharmacological experiments.

## Figures and Tables

**Table 1:** The ^1^H and ^13^C-NMR assignments of compound **1.**

Atom	C Shift	H Shift	H Multiplicity
1	44.6	1.27	m
		2.14	dd (12.7, 4.4)
2	68.0	3.58	m
3	82.4	2.82	d (9.7)
4	38.9		
5	48.8	1.11	m
6	23.0	2.05	m
7	123.8	5.57	d (5.2)
8	142.2		
9	149.2		
10	38.8		
11	117.2	5.30	br s
12	79.7	3.70	br s
13	45.3		
14	47.8		
15	33.0	1.74	m
		1.46	m
16	25.6	1.88	m
		1.49	m
17	46.2	1.44	m
18	13.9	0.50	s
19	22.3	0.97	s
20	38.2	1.75	m
21	73.8	3.91	dd (11.5, 4.3)
		2.95	t (11.1)
22	29.3	1.17	m
		1.30	m
23	28.6	1.54	m
		1.15	m
24	78.4	3.09	dd (9.8, 1.6)
25	72.3		
26	23.4	1.02	s
27	24.4	1.05	s
28	24.1	0.80	s
29	16.1	0.80	s
30	27.8	0.91	s

**Table 2:** The ^1^H and ^13^C-NMR assignments of compound **2.**

Atom	C Shift	H Shift	H Multiplicity
1	182.9		
2	39.1	2.16	t (7.6)
3	27.7	1.61	m
4	30.9	1.34	m
5	30.6	1.34	m
6	26.6	1.35	m
7	38.2	1.53	m
8	72.7	4.18	q (6.3)
9	148.7	6.24	dd (15.2, 5.8)
10	128.9	6.42	dd (15.2, 10.8)
11	144.6	7.27	dd (15.6, 10.8)
12	130.5	6.18	d (15.6)
13	204.0		
14	41.2	2.61	t (7.4)
15	25.4	1.60	m
16	32.7	1.31	m
17	23.7	1.34	m

## Supplementary Materials

Supplementary materials are available online: Figures S1–S39, Tables S1–S3.

## References

[1] Boa E. (2014). Non-Wood Forest Products 17 Wild edible fungi A global overview of their use and importance to people.

[2] Bessette A.E., Bessette A.R., Fischer D.W. (1997). Mushrooms of Northeastern North America.

[3] Ewald G. (2003). BLV-Bestimmungsbuch Pilze.

[4] De Bernardi M., Mellerio G., Vidari G., Vita-Finzi P., Fronza G., Kocor M., Pyrek J.S. (1981). Fungal metabolites IX. Triterpenes from *Naematoloma sublateritium*. J. Nat. Prod..

[5] Yaoita Y., Matsuki K., Iijima T., Nakano S., Kakuda R., Machida K., Kikuchi M. (2001). Studies on the Constituents of mushrooms. Part XII. New sterols and triterpenoids from four edible mushrooms. Chem. Pharm. Bull..

[6] Backens S., Steffan B., Steglich W., Zechlin L., Anke T. (1984). Antibiotics from Basidiomycetes, XIX. Naematolin and naematolone, two caryophyllane derivatives from cultures of Hypholoma species (Agaricales). Justus Liebigs Ann. Chem..

[7] Lee Y.R., Kim K.M., Jeon B.H., Choi J.W., Choi S. (2012). The *n*-butanol fraction of *Naematoloma sublateritium* suppresses the inflammatory response through downregulation of NF-κB in human endothelial cells. Int. J. Mol. Med..

[8] Lee Y.R., Jeon B.H., Choi S., Kim K.M. (2014). The hexane fraction of *Naematoloma sublateritium* extract suppresses the TNF-α-induced metastatic potential of MDA-MB-231 breast cancer cells through modulation of the JNK and p38 pathways. Int. J. Oncol..

[9] Liktor-Busa E., Kovács B., Urbán E., Hohmann J., Ványolós A. (2016). Investigation of Hungarian mushrooms for antibacterial activity and synergistic effects with standard antibiotics against resistant bacterial strains. Lett. Appl. Microbiol..

[10] Li H., Nam W.-S., Moon B., Lee C. (2014). Antioxidant activity and phenolic content of brick caps mycelium (*Naematoloma sublateritium*) extracts. Food Sci. Biotechnol..

[11] Ványolós A., Orbán-Gyapai O., Hohmann J. (2014). Xanthine Oxidase Inhibitory Activity of Hungarian Wild-Growing Mushrooms. Phytother. Res..

[12] Enesco H.E. (1993). Rotifers in aging research: Use of rotifers to test various theories of aging. Hydrobiologia.

[13] Snare D.J., Fields A.M., Snell T.W., Kubanek J. (2013). Lifespan extension of rotifers by treatment with red algal extracts. Exp. Gerontol..

[14] Snell T.W. (2014). Rotifers as models for the biology of aging. Int. Rev. Hydrobiol..

[15] Clément P., Amsellem J., Cornillac A.M., Luciani A., Ricci C. (1980). An ultrastructural approach to feeding behaviour in *Philodina roseola* and *Brachionus calycyflorus* (rotifers). Hydrobiologia.

[16] Hochberg R., Litvaitis M.K. (2000). Functional morphology of the muscles in Philodina sp. (*Rotifera: Bdelloidea*). Hydrobiologia.

[17] Olah Z., Bush A.I., Aleksza D., Galik B., Ivitz E., Macsai L., Janka Z., Karman Z., Kalman J., Datki Z. (2017). Novel in vivo experimental viability assays with high sensitivity and throughput capacity using a bdelloid rotifer. Ecotoxicol. Environ. Saf..

[18] Kleinwachter P., Luhmann U., Schlegel B., Heinze S., Hartl A., Kiet T.T., Grafe U. (1999). New fasciculol-type triterpene compounds from *Hypholoma fasciculare*. J. Basic Microbiol..

[19] Kim K.H., Moon E., Choi S.U., Kim S.Y., Lee K.R. (2013). Lanostane triterpenoids from the mushroom *Naematoloma fasciculare*. J. Nat. Prod..

[20] Ikeda M., Niwa G., Tohyama K., Sassa T., Miura Y. (1977). Structures of fasciculol C and its depsipeptides, new biologically active substances from *Naematoloma fasciculare*. Agric. Biol. Chem..

[21] Ikeda M., Sato Y., Izawa M., Sassa T., Miura Y. (1977). Structures of Fasciculol B and Its Depsipeptide, New Biologically Active Substances from *Naematoloma fasciculare*. Agric. Biol. Chem..

[22] Takahashi A., Kusano G., Ohta T., Ohizumi Y., Nozoe S. (1989). Fasciculic Acids A, B and C as Calmodulin Antagonists from the Mushroom *Naematoloma fasciculare*. Chem. Pharm. Bull..

[23] Clericuzio M., Piovano M., Chamy M.C., Garbarino J.A., Milanesio M., Viterbo D., Vidari G., Finzi P.V. (2004). Structural characterisation of metabolites from Pholiota spumosa (Basidiomycetes). Croat. Chem. Acta.

[24] Mansoor T.A., Hong J., Lee C.-O., Bae S.-J., Im K.S., Jung J.H. (2005). Cytotoxic sterol derivatives from a marine sponge *Homaxinella* sp.. J. Nat. Prod..

[25] Kiss T., Mácsai L., Csupor D., Datki Z.L. (2017). In vivo screening of diterpene alkaloids using bdelloid rotifer assays. Acta Biol. Hung..

