# Supplementary material for: Triterpenes from the Mushroom Hypholoma lateritium: Isolation, Structure Determination and Investigation in Bdelloid Rotifer Assays
Source: Molecules. 2019 Jan 15;24(2):301. doi: 10.3390/molecules24020301 (PMC6359218; doi:10.3390/molecules24020301)
Supplement: Supplementary file 1 [file molecules-24-00301-s001.pdf]

Triterpenes from the Mushroom *Hypholoma lateritium*: Isolation, Structure Determination, and Investigation in Bdelloid Rotifer Assays

Bayar Chuluunbaatar[1], Zoltán Béni[2], Miklós Dékány[2], Bernadett Kovács[1], András Sárközy[1], Zsolt Datki [3], Lilla Mácsai[3], János Kálmán[3], Judit Hohmann[1,4], Attila Ványolós[1]*

[1] *Department of Pharmacognosy, University of Szeged, Eötvös u. 6, H-6720 Szeged, Hungary; ch_bayaraa@pharmacognosy.hu, kovacs.bernadett@pharmacognosy.hu, sarkozy@pharmacognosy.hu*

[2] *Spectroscopic Research, Gedeon Richter Plc., Gyömrői út 19-21, H-1103 Budapest, Hungary; z.beni@richter.hu, M.Dekany@richter.hu*

[3] *Department of Psychiatry, Faculty of Medicine, University of Szeged, Kálvária sgt. 57, H-6725 Szeged, Hungary, datkizsolt@gmail.com, macsai.lilla@gmail.com, kalman.janos@med.u-szeged.hu*

[4]*Interdisciplinary Centre for Natural Products, University of Szeged, Eötvös u. 6, H-6720 Szeged, Hungary; hohmann@pharm.u-szeged.hu*

*Correspondence: vanyolosa@pharmacognosy.hu; Tel.: +36-62-54-6455

TABLE OF CONTENTS

NMR and MS spectra and spectral data of compound 1 .... **4**

Figure S1. Full HRMS spectrum of compound **1** 4

Figure S2. HR-MS/MS spectrum of compound **1** 5

Figure S3. ^{1}H NMR spectrum of compound **1** 5

Figure S4. ^{13}C NMR spectrum of compound **1** 5

Figure S5. ROESY spectrum of compound **1** 6

Figure S6. COSY spectrum of compound **1** 6

Figure S7. HSQC spectrum of compound **1** 7

Figure S8. HMBC spectrum of compound **1** 7

NMR and MS spectra and spectral data of compound 2 .... **8**

Figure S9. Full HRMS spectrum of compound **2** 8

Figure S10. HR-MS/MS spectrum of compound 2 9

Figure S11. ^{1}H NMR spectrum of compound **2** 9

Figure S12. ^{13}C NMR spectrum of compound **2.** 10

Figure S13. HMBC spectrum of compound **2** 10

Figure S14. HSQC spectrum of compound **2** 11

Figure S15. COSY spectrum of compound **2** 11

NMR and MS spectra and spectral data of compound 5 .... **12**

Figure S16. Full HRMS spectrum of compound **5** 12

Figure S17. HR-MS/MS spectrum of compound **5** 12

Table S1. 1H and 13C NMR assignments of compound **5** 13

Figure S18. ^{1}H spectrum of compound **5** 14

Figure S19. ^{13}C spectrum of compound **5** 14

NMR and MS spectra and spectral data of compounds 8, 9 and 10 .... **15**

Table S2. ^{1}H and ^{13}C NMR assignments of compounds **8**, **9** and **10** 16

Figure S20. Full HRMS spectrum of compound **8** 17

Figure S21. HR-MS/MS spectrum of compound **8** 18

Figure S22. ^{1}H spectrum of compound **8** 18

Figure S23. ^{13}C NMR spectrum of compound **8** 19

Figure S24. Full HRMS spectrum of compound **9** 19

Figure S25. HR-MS/MS spectrum of compound **9** 20

Figure S26. ^{1}H spectrum of compound **9** 20

Figure S27. ^{13}C spectrum of compound **9** 21

Figure S28. Full HRMS spectrum of compound **10** 22

Figure S29. HR-MS/MS spectrum of compound **10** 22

Figure S30. ^{1}H spectrum of compound **10** 23
Figure S31. ^{13}C spectrum of compound **10** 23
NMR and MS spectra and spectral data of compound 11 24
Figure S32. Full HRMS spectrum of compound **11** 24
Figure S33. HR-MS/MS spectrum of compound **11** 24
Table S3. NMR assignments of compound **11** 25
Figure S34. ^{1}H spectrum of compound **11** 26
Figure S35. ^{13}C spectrum of compound **11** 26
NMR and MS spectra and spectral data of compound 12 27
Figure S36. Full HRMS spectrum of compound **12** 27
Figure S37. HR-MS/MS spectrum of compound **12** 27
Figure S38. ^{1}H spectrum of compound **12** 28
Figure S39. ^{13}C spectrum of compound **12** 28

NMR and MS Spectra and Spectral Data of Compound 1

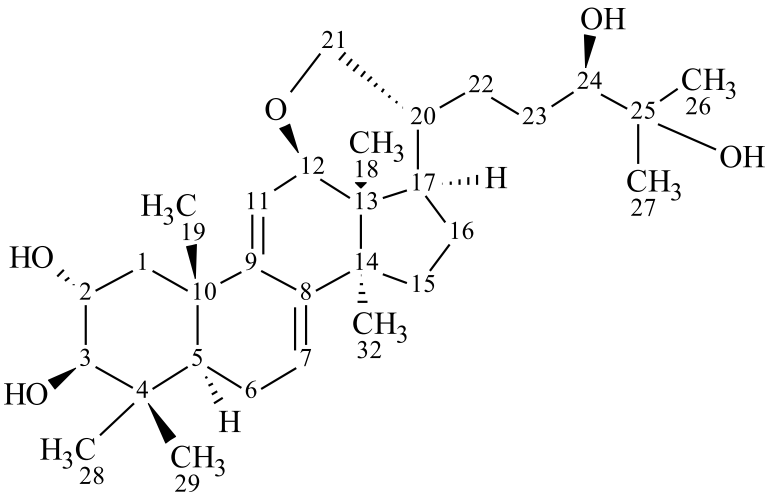

HRMS: (2M+2Na)/2=511.33967 (delta=0.5 ppm; $C_{60}H_{96}O_{10}Na_2$ /*z*=*2*/). HR-ESI-MS-MS (CID=55%; rel. int. %): 571(100); 553(28); 535(6); 499(14); 471(10); 453(70).

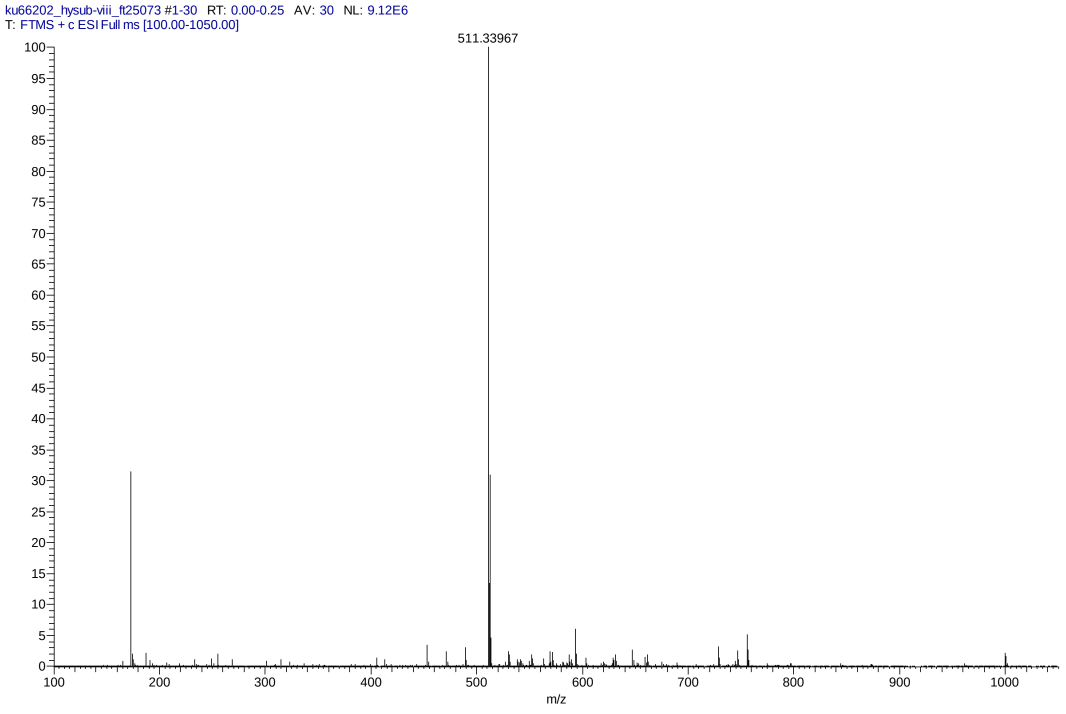

Figure S1. Full HRMS spectrum of compound **1**.

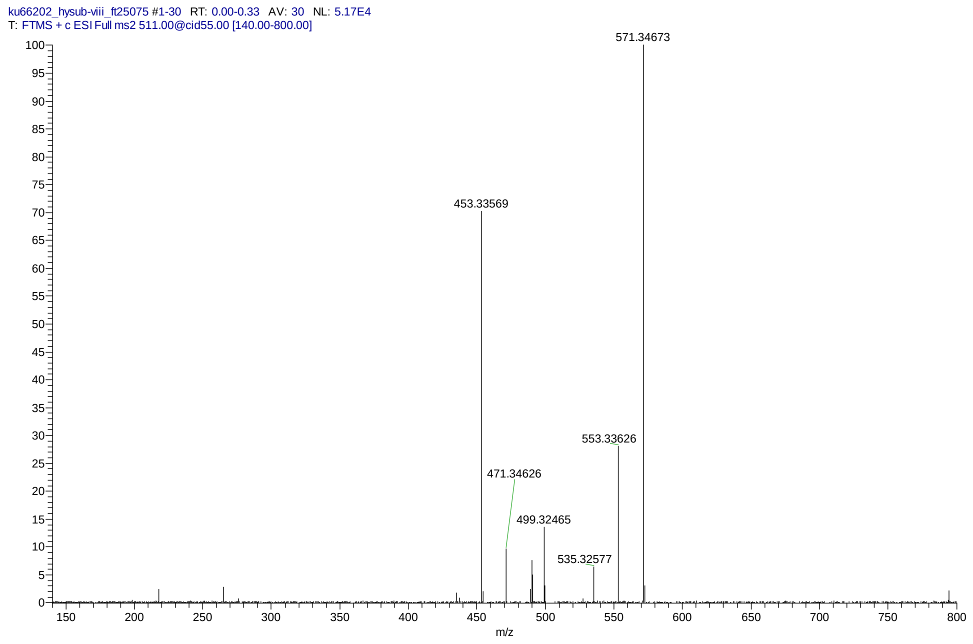

Figure S2. HR-MS/MS spectrum of compound **1**.

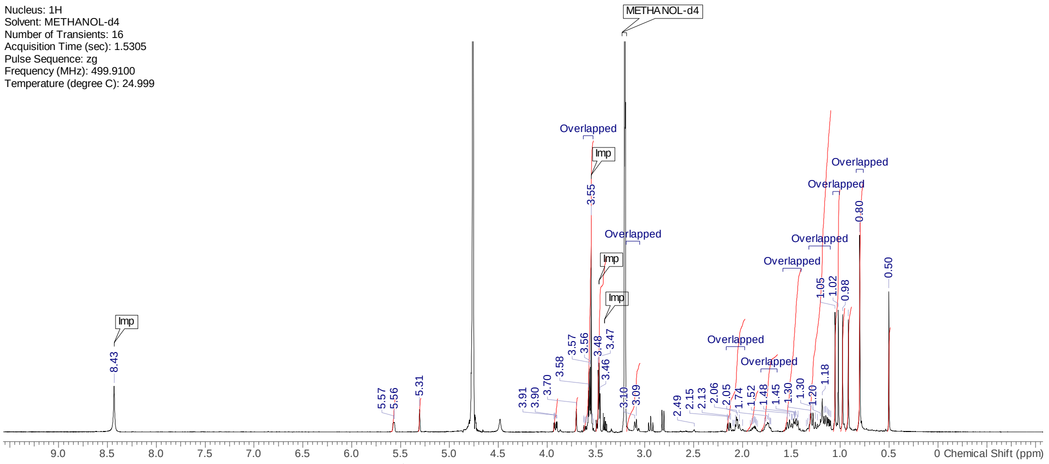

Figure S3. The ^{1}H NMR spectrum of compound **1**.

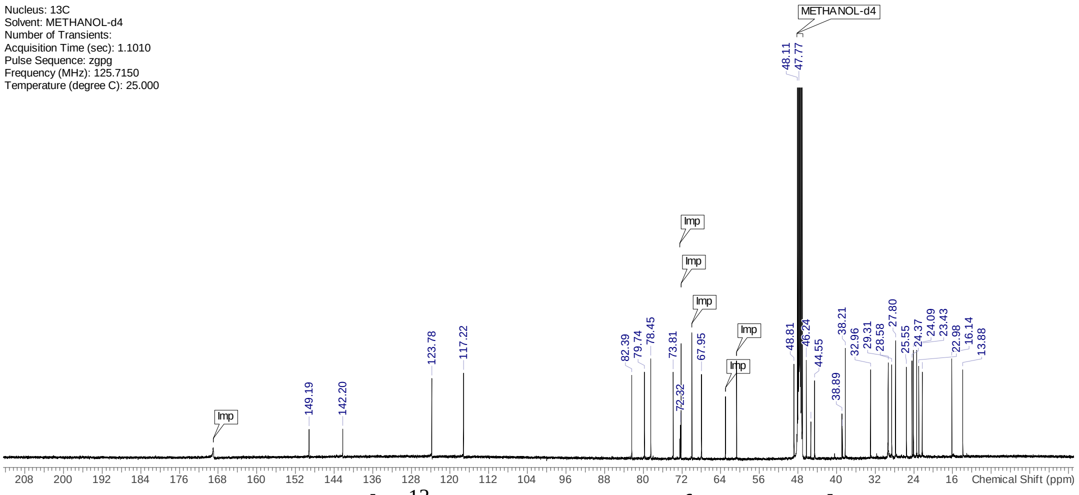

Figure S4. The ^{13}C NMR spectrum of compound **1**.

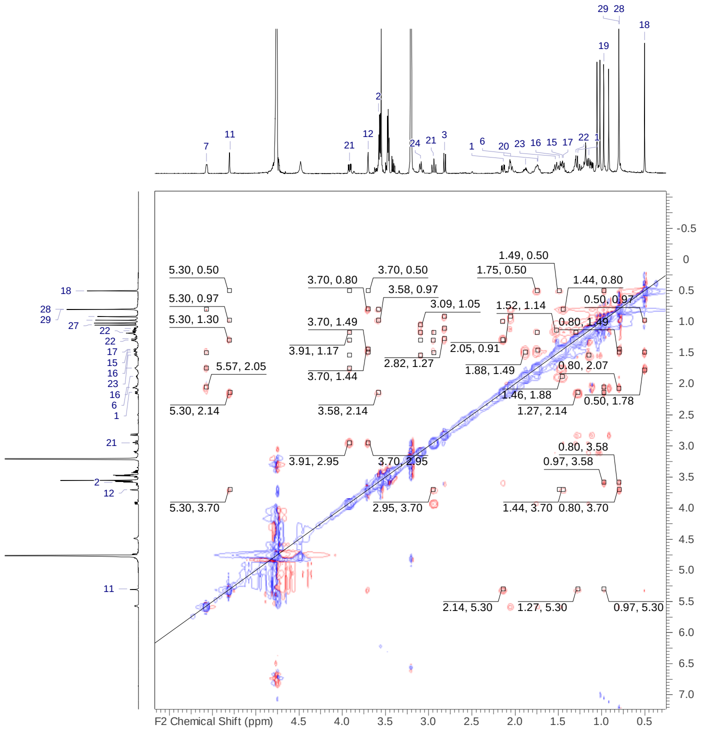

Figure S5. ROESY spectrum of compound **1**.

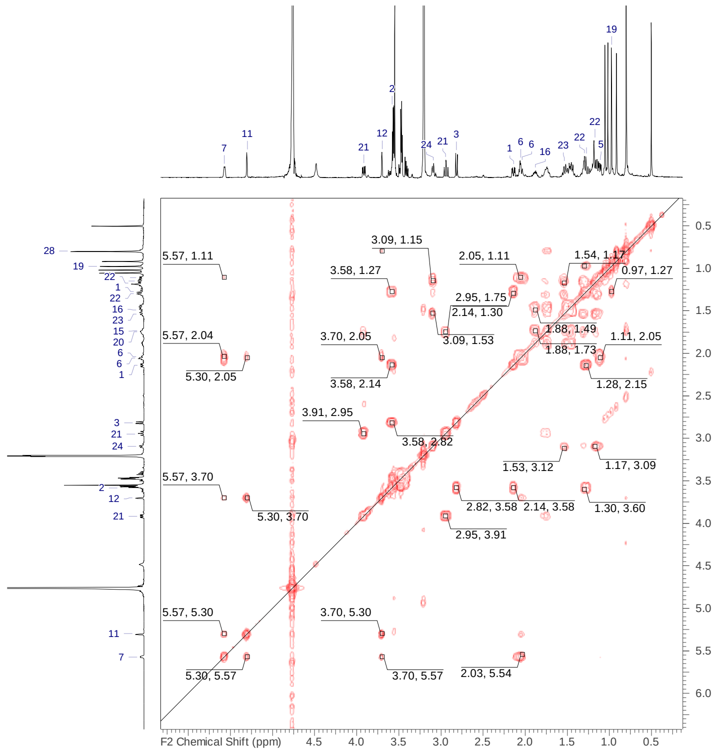

Figure S6. COSY spectrum of compound **1**,

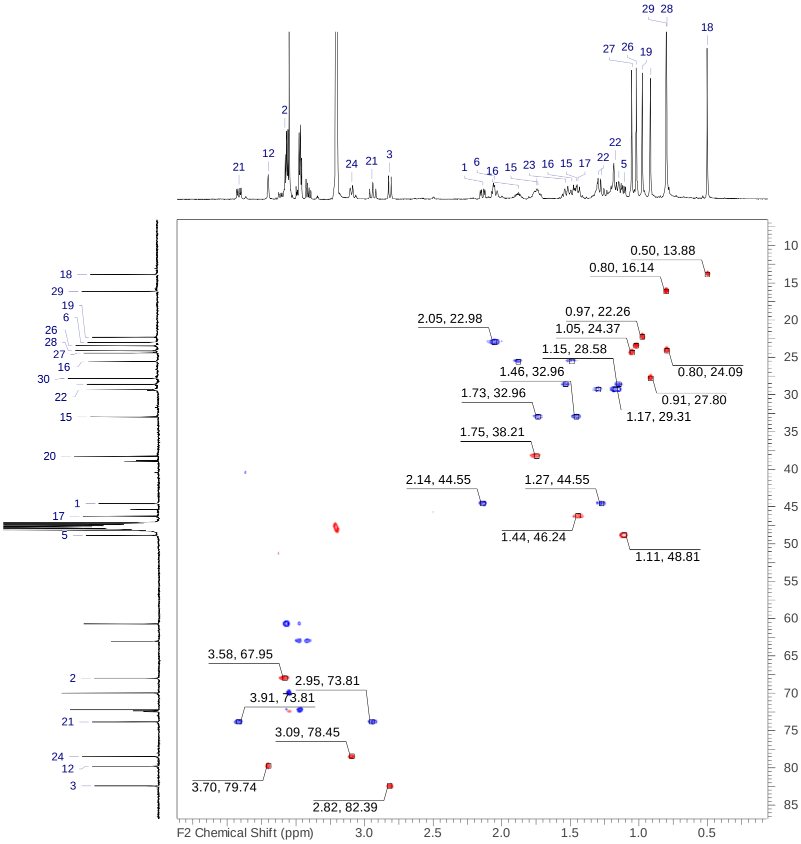

Figure S7. HSQC spectrum of compound **1**.

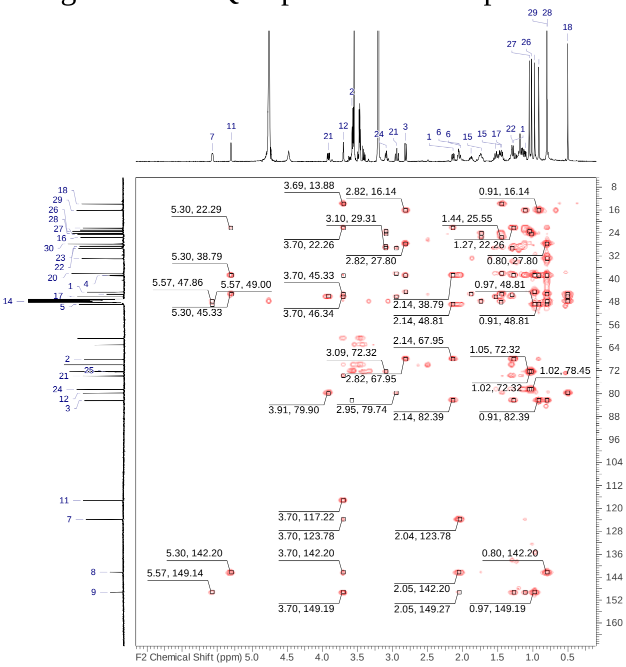

Figure S8. HMBC spectrum of compound **1**.

NMR and MS Spectra and Spectral Data of Compound 2

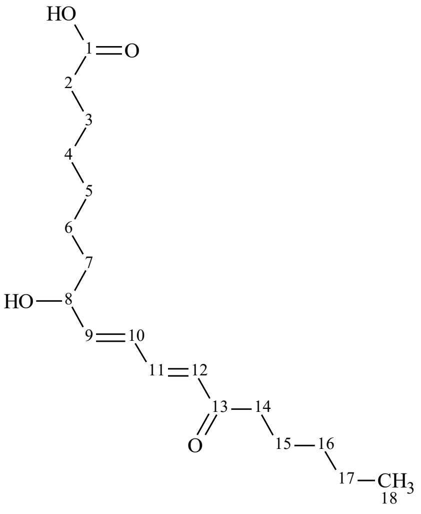

HRMS: M-H=309.20673 (delta=-1.3 ppm; $C_{18}H_{29}O_4$). HR-ESI-MS-MS (CID=55%; rel. int. %): 291(100); 209(8); 195(21); 171(4).

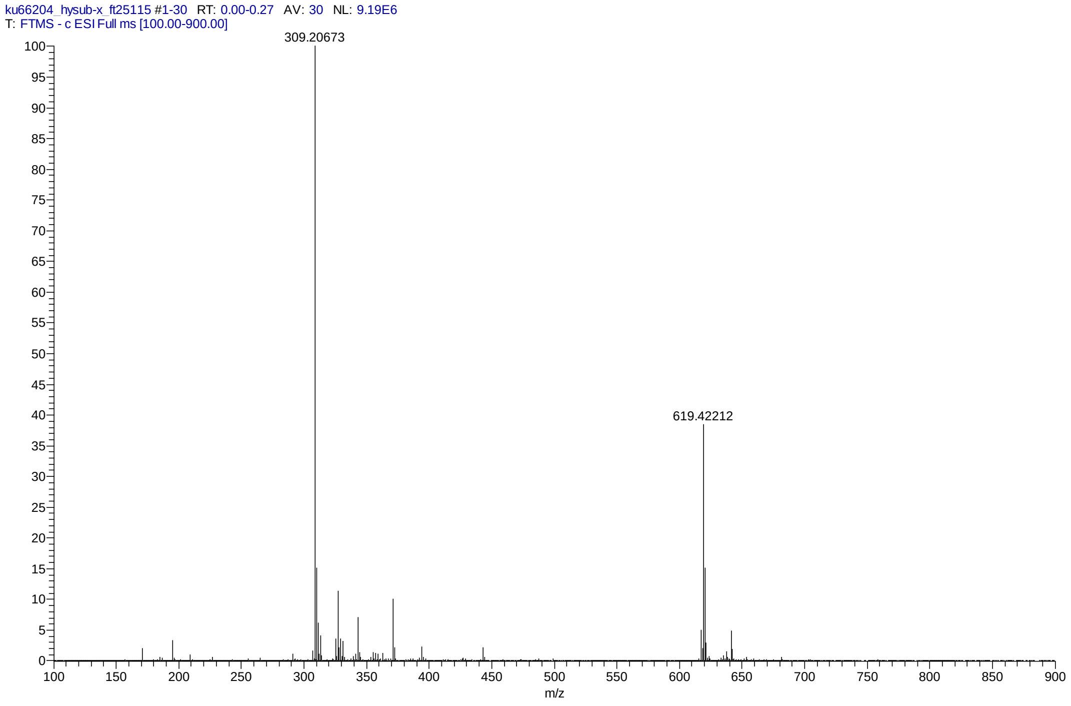

Figure S9. Full HRMS spectrum of compound **2**.

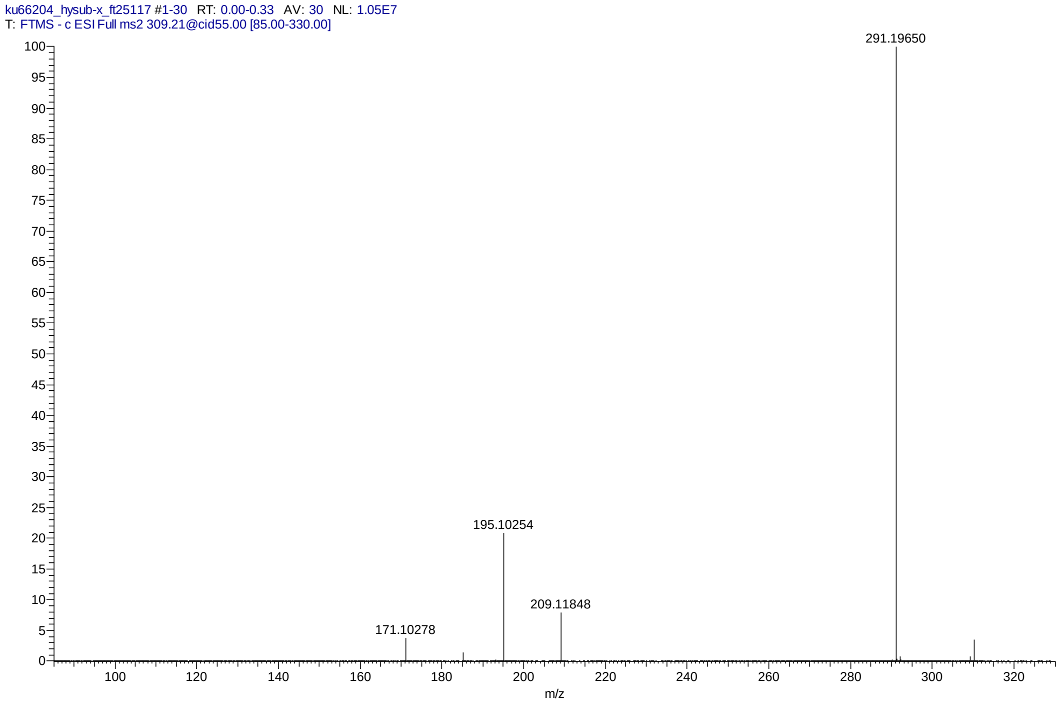

Figure S10. HR-MS/MS spectrum of compound **2**.

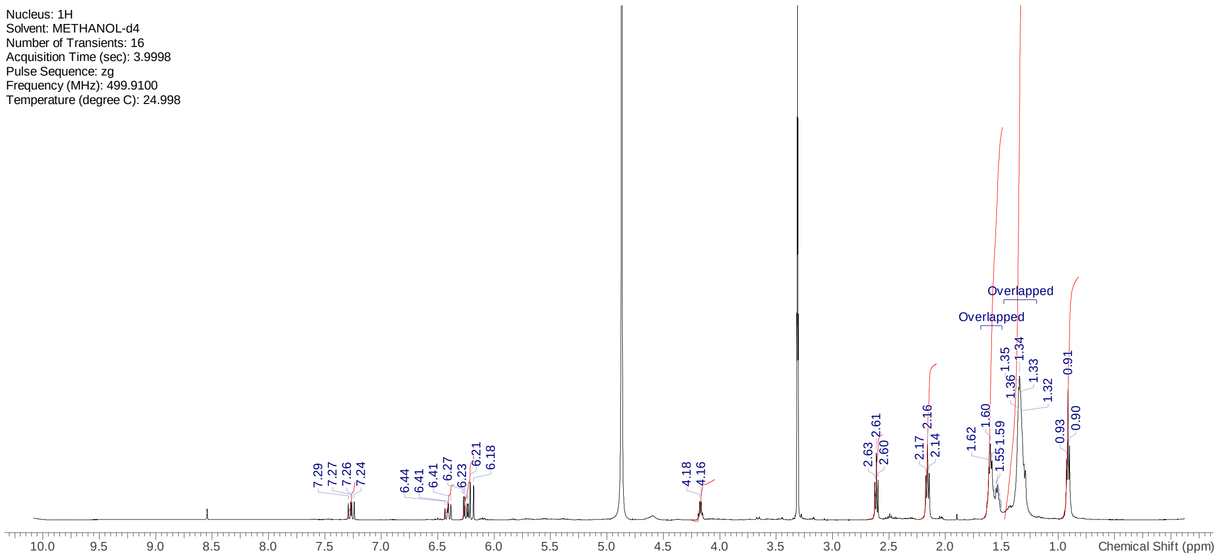

Figure S11. The ^{1}H NMR spectrum of compound **2**.

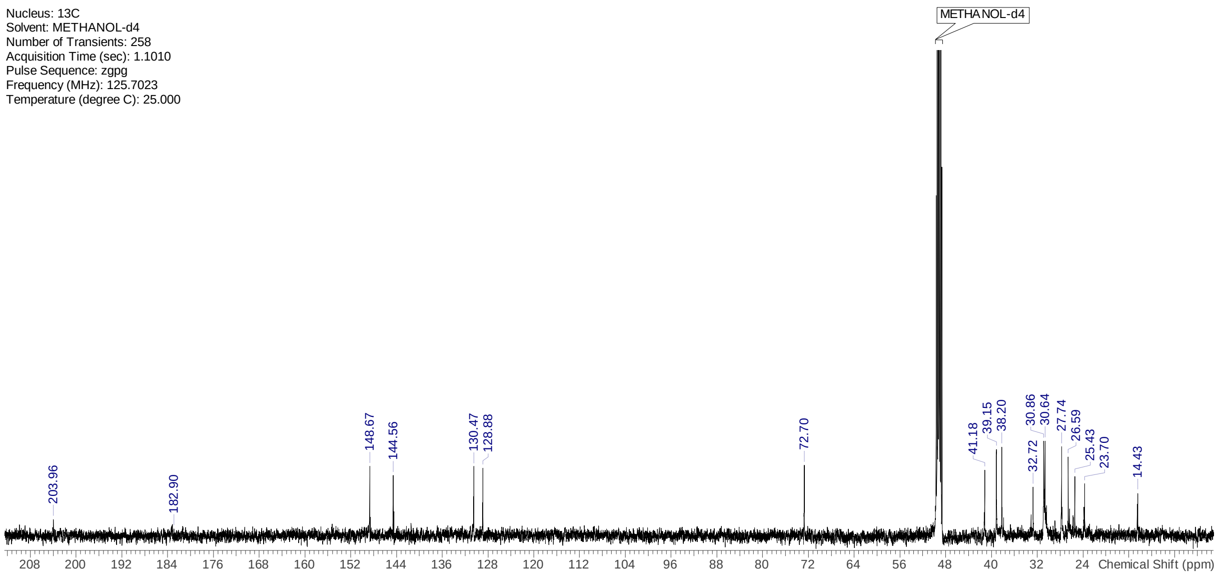

Figure S12. The ^{13}C NMR spectrum of compound **2**.

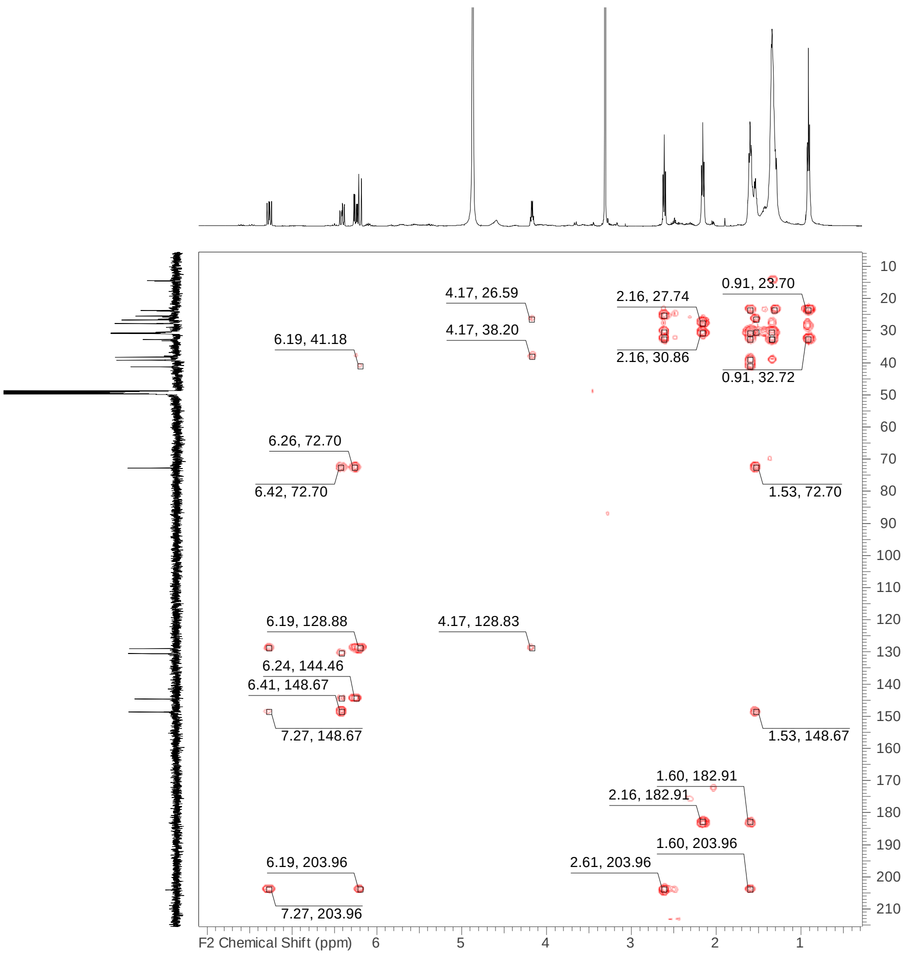

Figure S13. HMBC spectrum of compound **2**.

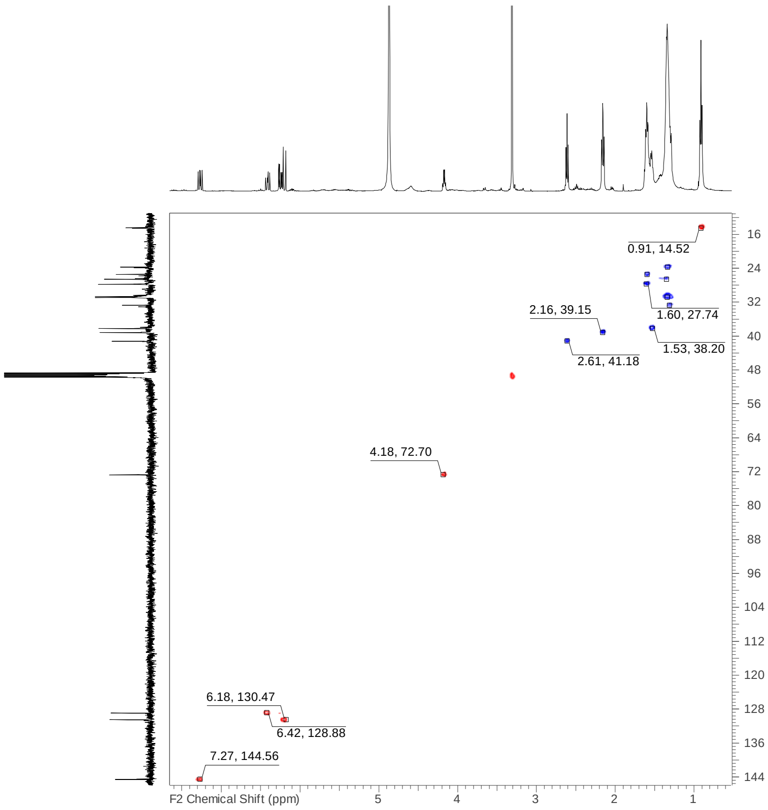

Figure S14. HSQC spectrum of compound **2**.

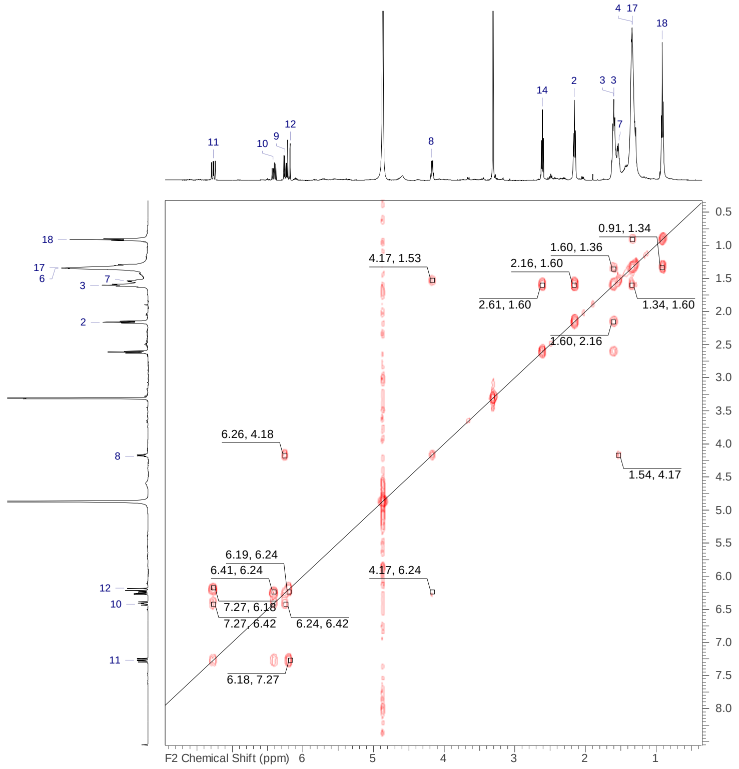

Figure S15. COSY spectrum of compound **2**.

NMR and MS Spectra and Spectral Data of Compound 5

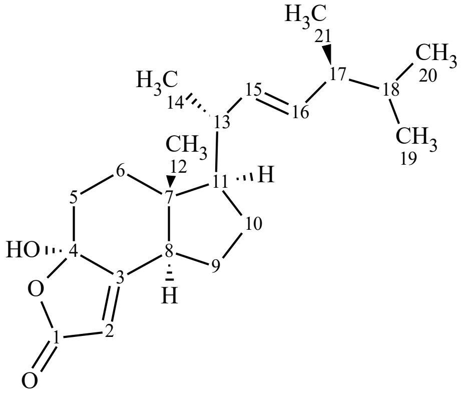

HRMS: M+H=333.24277 (delta=1.0 ppm; $C_{21}H_{33}O_3$). HR-ESI-MS-MS (CID=45%; rel. int. %): 315(100).

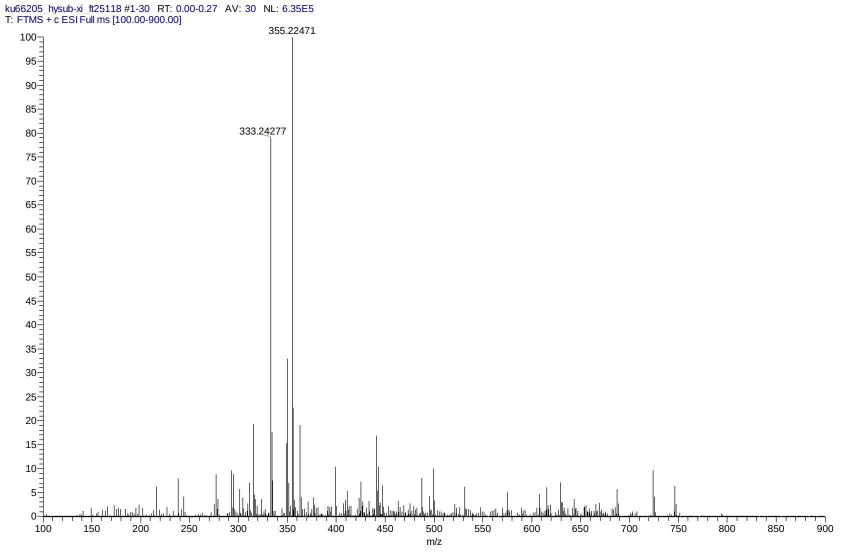

Figure S16. Full HRMS spectrum of compound **5**.

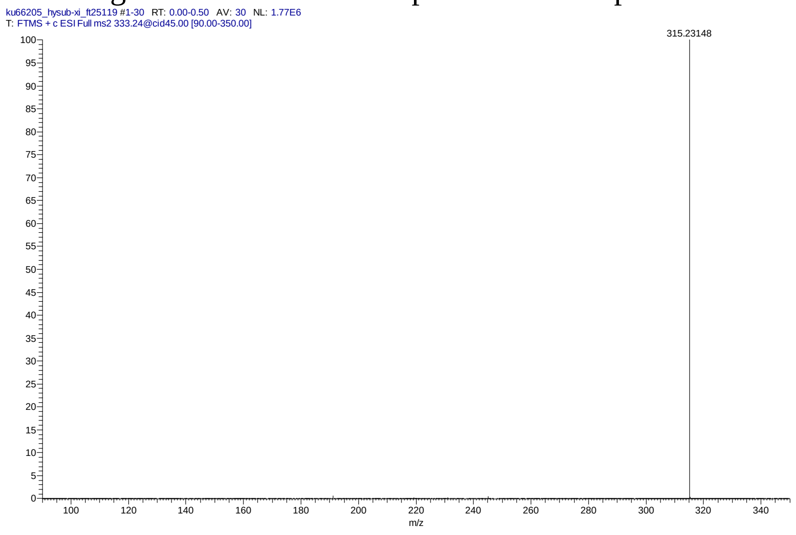

Figure S17. HR-MS/MS spectrum of compound **5**.

Table S1. The 1H and 13C NMR assignments of compound **5**.

Atom#	C Shift	H Shift	multiplicity (J in Hz)
1	173.8	-	-
2	112.7	5.68	d (1.8)
3	173.3	-	-
4	107.3	-	-
5	36.3	1.80	td (13.6, 4.8)
5	36.3	2.23	ddd (13.6, 4.1, 2.7)
6	36.6	1.61	m
6	36.6	1.97	m
7	50.0	-	-
8	51.9	2.66	ddd (12.4, 7.1, 1.6)
9	22.4	1.72	m
9	22.4	1.60	m
10	30.2	1.93	m
10	30.2	1.50	m
11	56.9	1.52	m
12	12.2	0.65	s
13	41.7	2.09	m
14	21.7	1.06	d (6.64)
15	136.6	5.22	dd (15.2, 8.5)
16	134.0	5.29	dd (15.4, 7.6)
17	44.5	1.87	m
18	34.5	1.49	m
19	20.6	0.87	d (6.8)
20	20.2	0.85	d (6.8)
21	18.3	0.95	d (6.8)

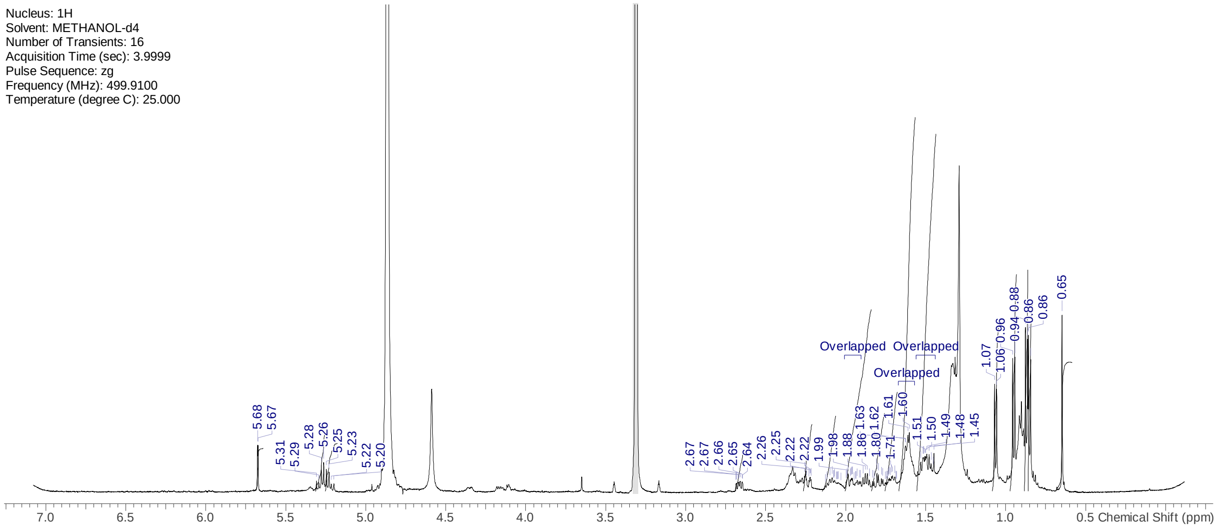

Figure S18. The ^{1}H spectrum of compound **5**.

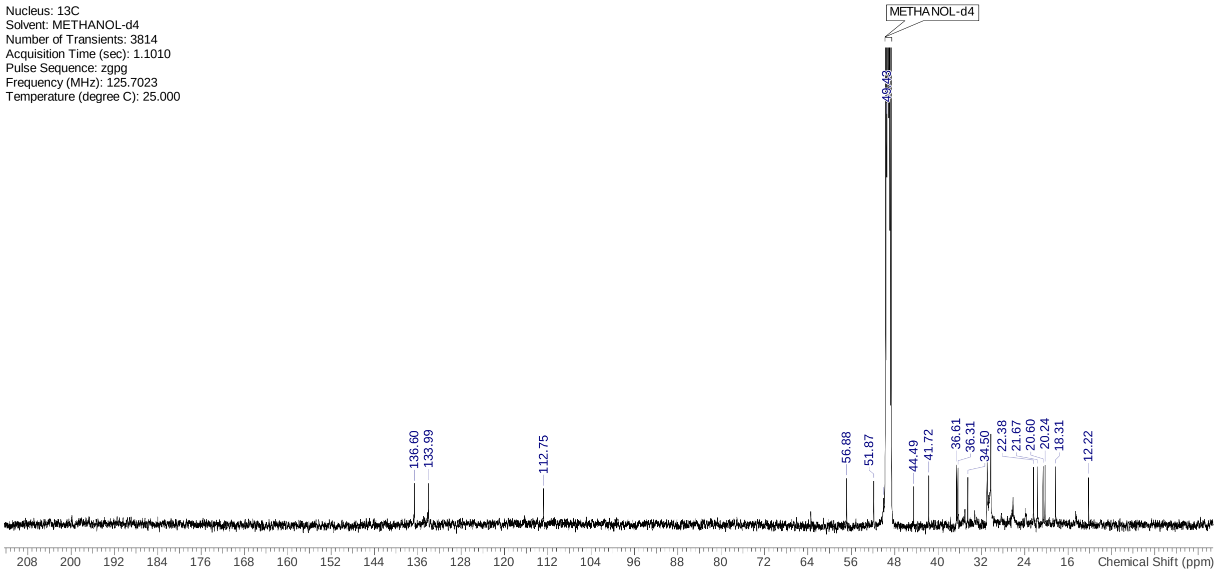

Figure S19. The ^{13}C spectrum of compound **5**.

NMR and MS Spectra and Spectral Data of Compounds 8, 9, and 10

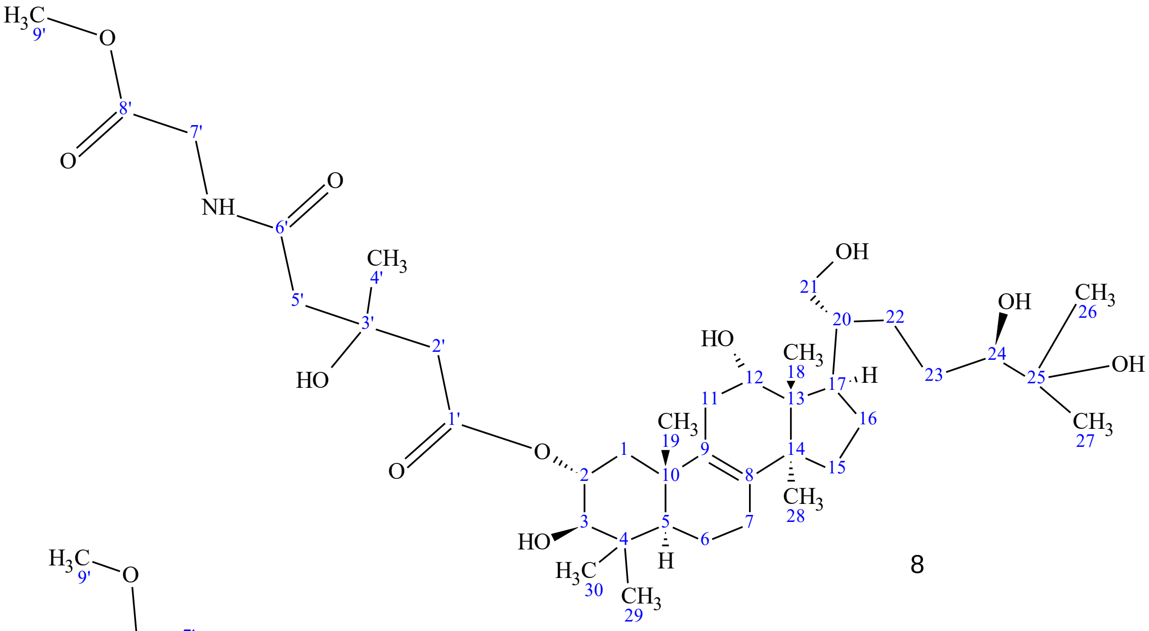

8

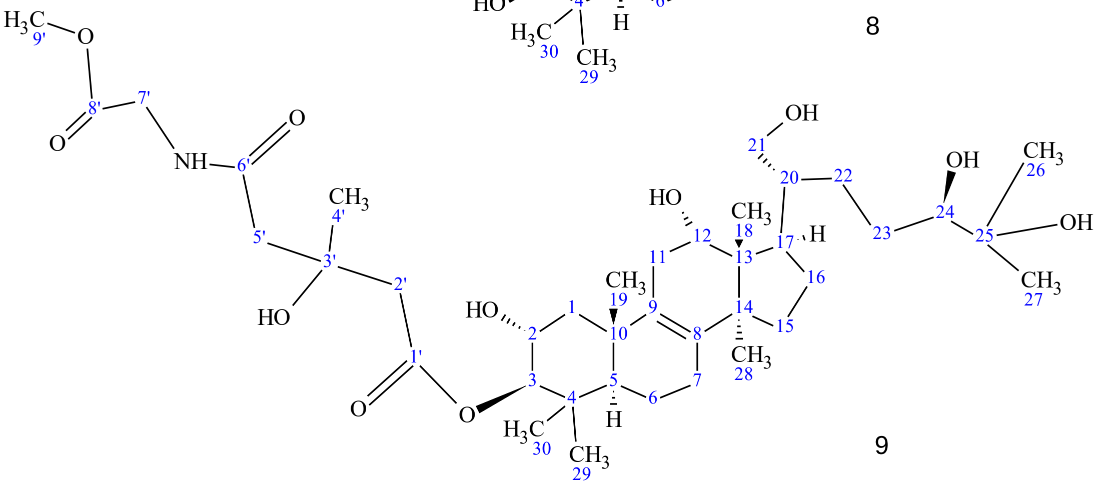

9

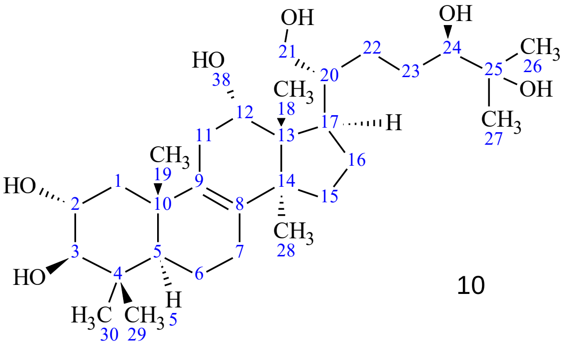

10

Table S2. The ^{1}H and ^{13}C NMR assignments of compounds **8**, **9**, and **10**

Trivial Name		Compound **9**			Compound **8**			Compound **10**		
		Fasciculol E			Fasciculol F			Fasciculol C		
Atom#		C Shift	H Shift	Multiplicity (J in Hz)	C Shift	H Shift	Multiplicity (J in Hz)	C Shift	H Shift	Multiplicity (J in Hz)
1	β	44.9	2.12	m	41.9	2.11	dd (12.3, 4.4)	45.0	2.05	m
1	α	44.9	1.26	m	41.9	1.24	m	45.0	1.16	m
2		68.2	3.83	m	74.5	5.03	ddd (11.7, 10.1, 4.4)	70.0	3.64	ddd (11.6, 9.7, 4.4)
3		86.0	4.58	d (9.9)	81.0	3.21	d (10.1)	84.4	2.93	d (9.7)
4		40.2	-	-	40.8	-	-	40.5	-	
5		52.0	1.27	m	52.0	1.21	m	52.1	1.16	m
6	α	19.4	1.58	m	19.4	1.76	m	19.5	1.73	m
6	β	19.4	1.74	m	19.4	1.58	m	19.5	1.56	m
7		27.6			27.7	2.13	m	27.7	2.10	m
7		27.6	2.12	m	27.7	2.07	m	27.7		
8		136.7			136.8			136.5		
9		134.2			134.0			134.5		
10		39.3			39.4			39.3		
11	β	33.4	2.76	m	33.3	2.71	m	33.4	2.03	m
11	α	33.4	2.05	m	33.3	1.96	m	33.4	2.75	m
12		74.5	4.03	d (8.1)	74.4	4.01	dd (8.9, 1.3)	74.5	4.02	d (7.8)
13		51.2			51.3			51.4		
14		51.4			51.2			51.2		
15	α	33.0	1.77	m	33.0	1.21	m	33.0	1.21	m
15	β	33.0	1.22	m	33.0	1.75	m	33.0	1.76	m
16	β	29.0	1.46	m	29.0	2.08	m	29.0	1.44	m
16	α	29.0	2.10	m	29.0	1.42	m	29.0	2.10	m
17		39.4	2.44	q(9.8)	39.3	2.43	q (9.6)	39.4	2.44	q (9.6)
18		17.5	0.65	s	17.5	0.64	s	17.5	0.65	s
19		20.5	1.09	s	20.3	1.12	s	20.5	1.06	s
20		44.4	1.40	m	44.4	1.39	m	44.4	1.38	m
21		62.1	3.72	m	62.0	3.70	m	62.1	3.71	m
21		62.1	3.80	m	62.0	3.79	dd (11.6, 2.6)	62.1	3.79	dd (11.3, 2.5)
22		28.2	1.46	m	28.2	1.45	m	28.2	1.43	m
22		28.2	1.68	m	28.2	1.67	m	28.2	1.69	m
23		29.5	1.42	m	29.5	1.42	m	29.5	1.43	m
23		29.5	1.56	m	29.5	1.56	m	29.5	1.56	m
24		79.5	3.26	dd (10.1, 1.8)	79.5	3.26	dd (10.3, 1.7)	79.6	3.26	dd (10.0, 1.7)
25		74.0			74.0			74.0		
26		25.0	1.14	s	25.0	1.14	s	25.0	1.14	s
27		25.9	1.17	s	25.9	1.17	s	25.9	1.17	s
28		24.3	1.09	s	24.3	1.07	m	24.3	1.07	s
29		18.1	0.92	s	17.3	0.89	s	17.4	0.84	s
30		29.2	0.92	s	29.1	1.07	s	29.2	1.03	s
1'		173.3			172.8					
2'		47.0	2.74	s	47.2	2.68	AB			
3'		71.7			71.6					

4'	28.1	1.42	s	28.1	1.40	s
5'	47.4	2.60	s	47.3	2.60	s
6'	174.2			174.2		
7'	172.0			42.0	3.97	AB
8'	41.9	3.97	AB	172.0		
9'	52.8	3.73	s	52.8	3.73	s

NMR and MS Spectra and Spectral Data of Compound **8**

HRMS: M+H=724.46289 (delta=-0.2 ppm; $C_{39}H_{66}O_{11}N$). HR-ESI-MS-MS (CID=35%; rel. int. %): 706(100); 688(31); 670(40); 652(7); 634(2).

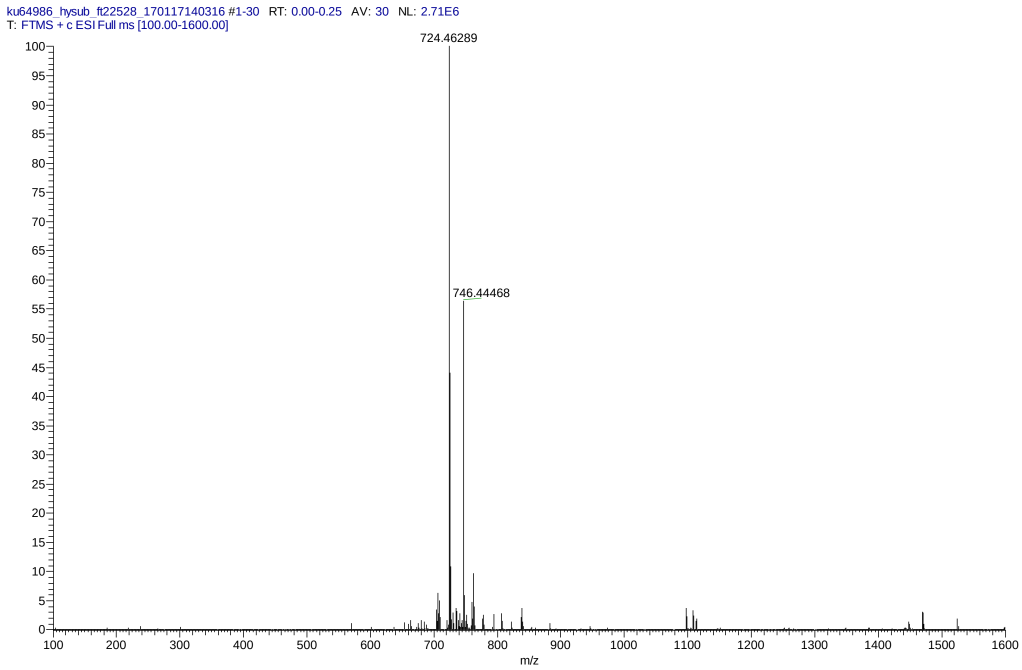

Figure S20. Full HRMS spectrum of compound **8**.

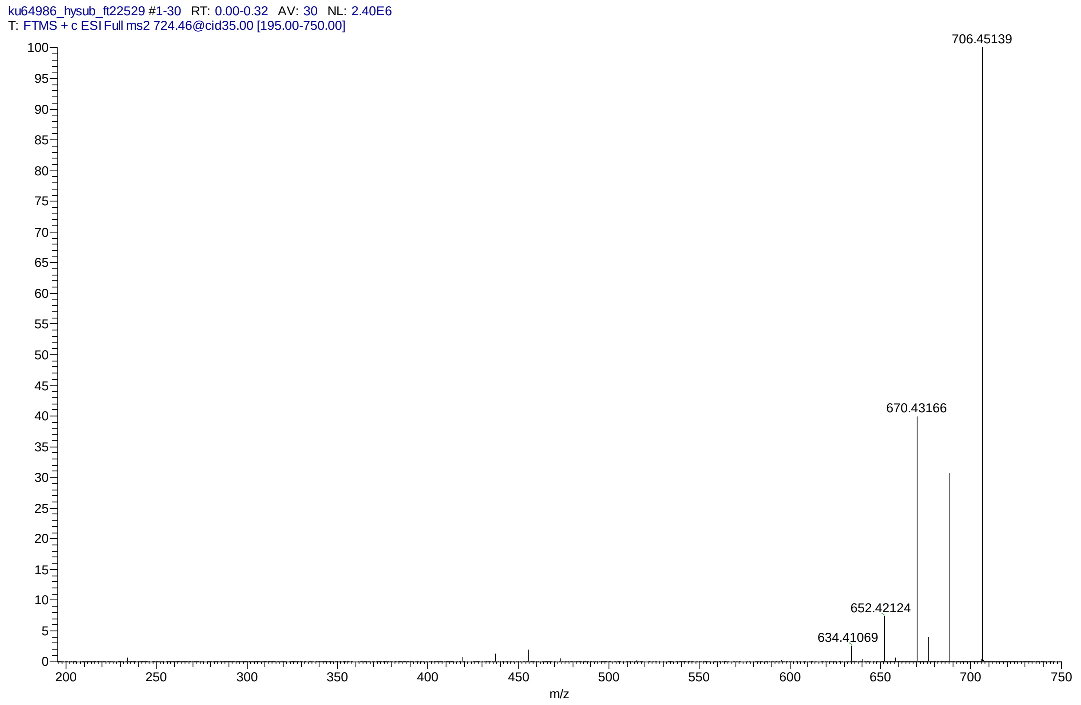

Figure S21. HR-MS/MS spectrum of compound **8**.

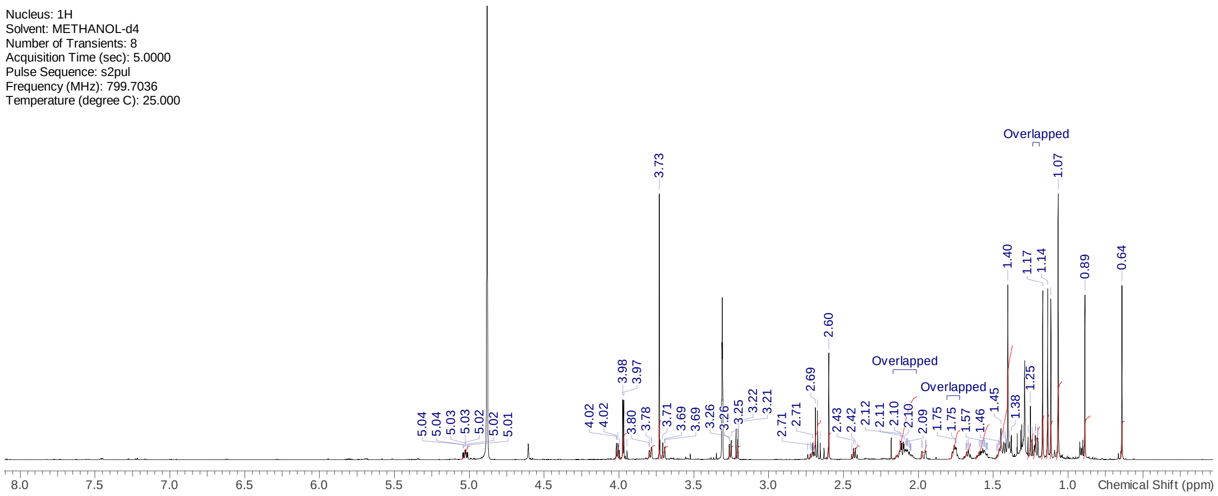

Figure S22. The ^{1}H spectrum of compound **8**.

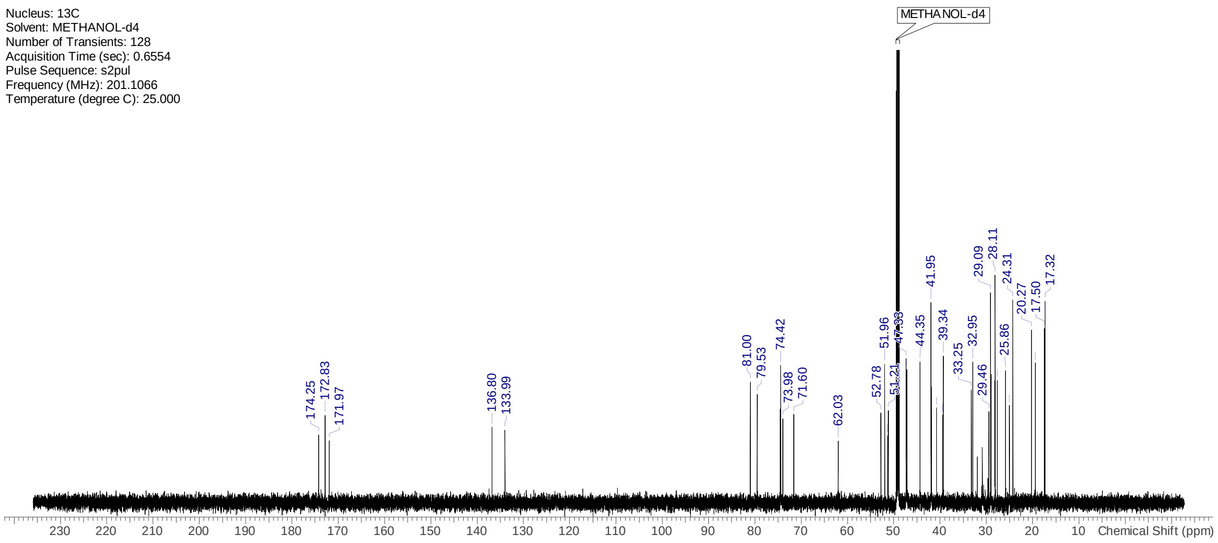

Figure S23. The ^{13}C NMR spectrum of compound **8**.

NMR and MS Spectra and Spectral Data of Compound **9**

HRMS: M+H=724.46357 (delta=0.7 ppm; $C_{39}H_{66}O_{11}N$). HR-ESI-MS-MS (CID=45%; rel. int. %): 688(100); 670(41); 652(10); 473(22); 455(33); 437(40); 419(25); 234(6).

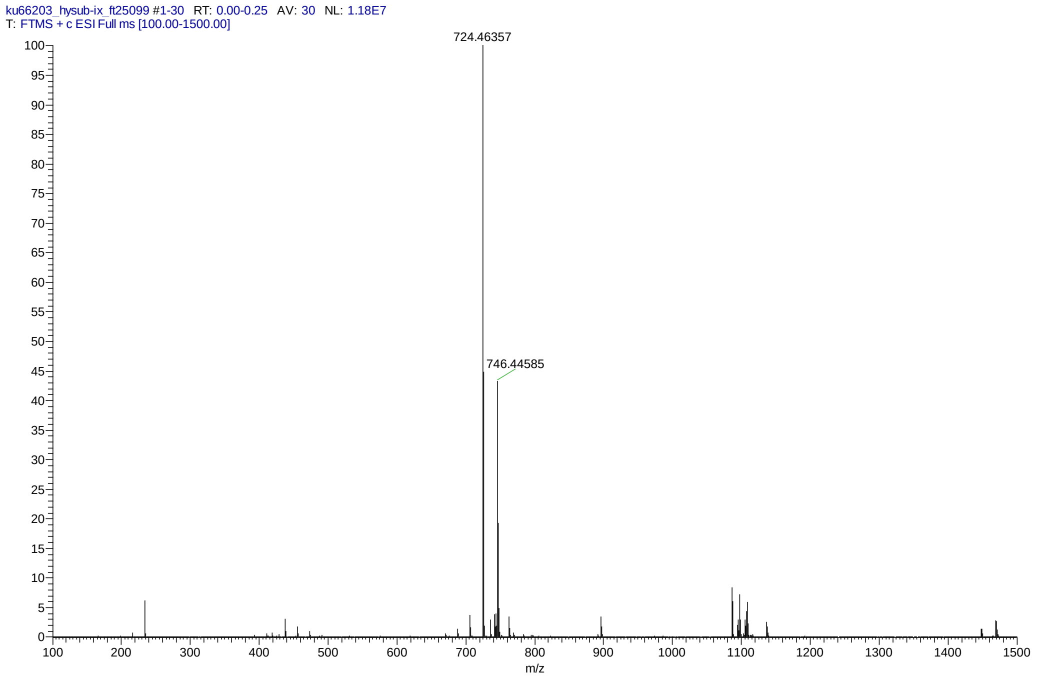

Figure S24. Full HRMS spectrum of compound **9**.

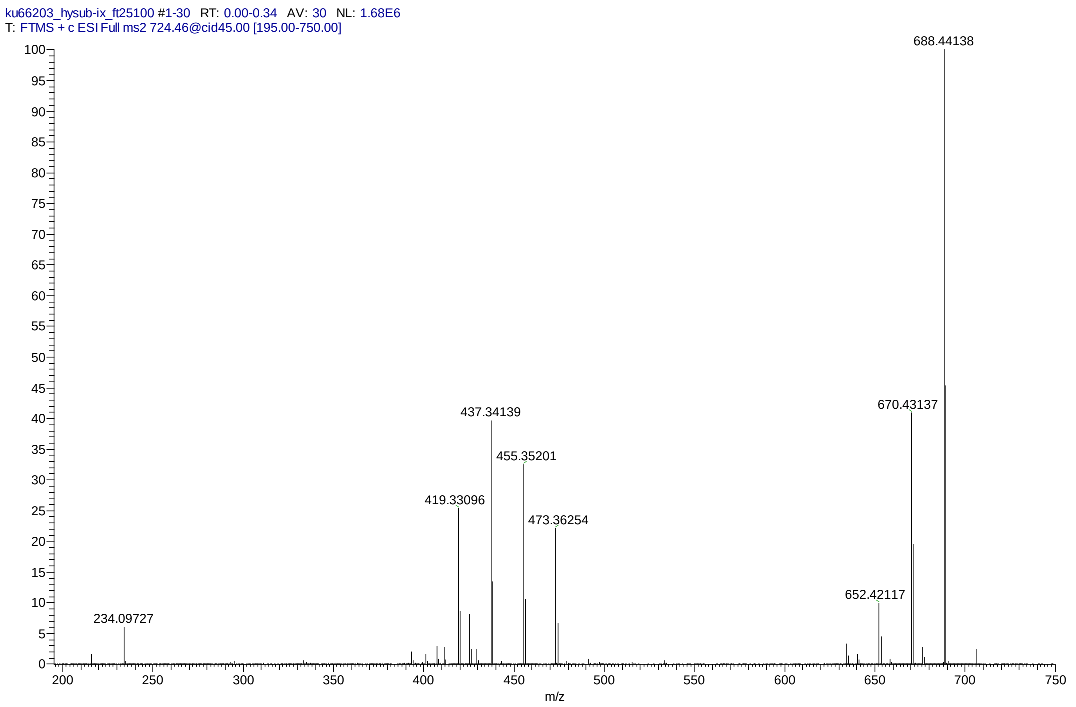

Figure S25. HR-MS/MS spectrum of compound **9**.

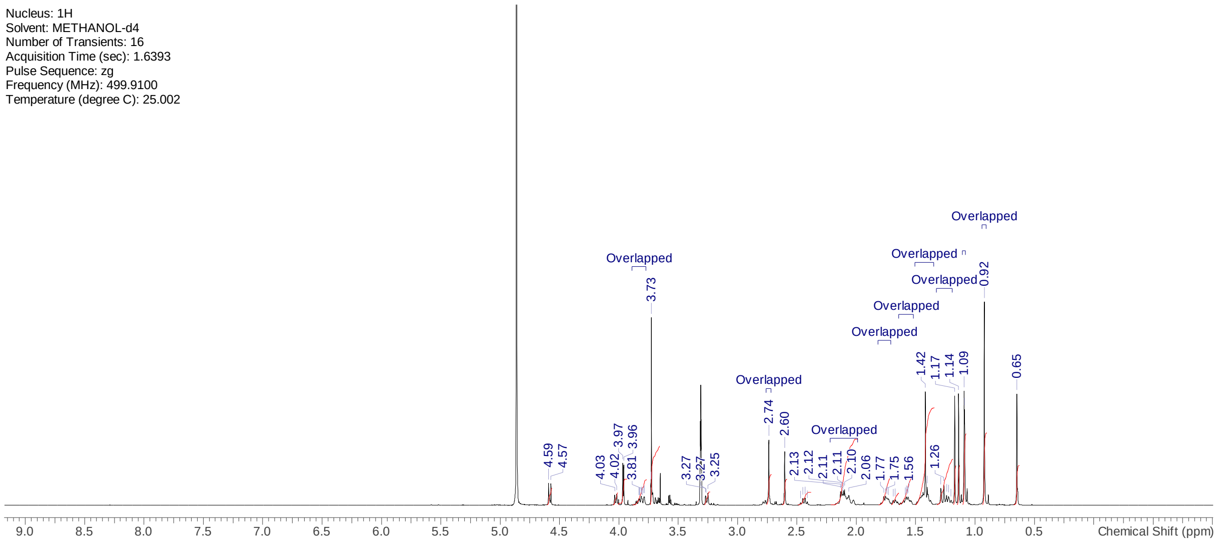

Figure S26. The ^{1}H spectrum of compound **9**.

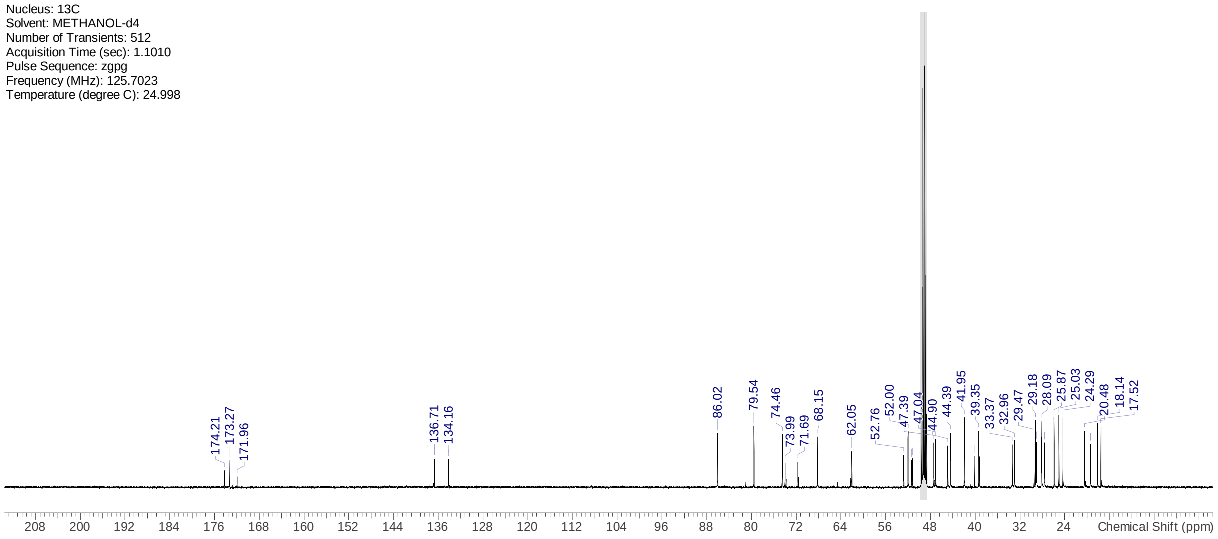

Figure S27. The ^{13}C spectrum of compound **9**.

NMR and MS Spectra and Spectral Data of Compound **10**

HRMS: M+H=509.38356 (delta=-0.2 ppm; $C_{30}H_{53}O_6$). HR-ESI-MS-MS (CID=35%; rel. int. %): 491(22); 473(100); 461(19); 455(94); 443(5); 437(15); 419(4).

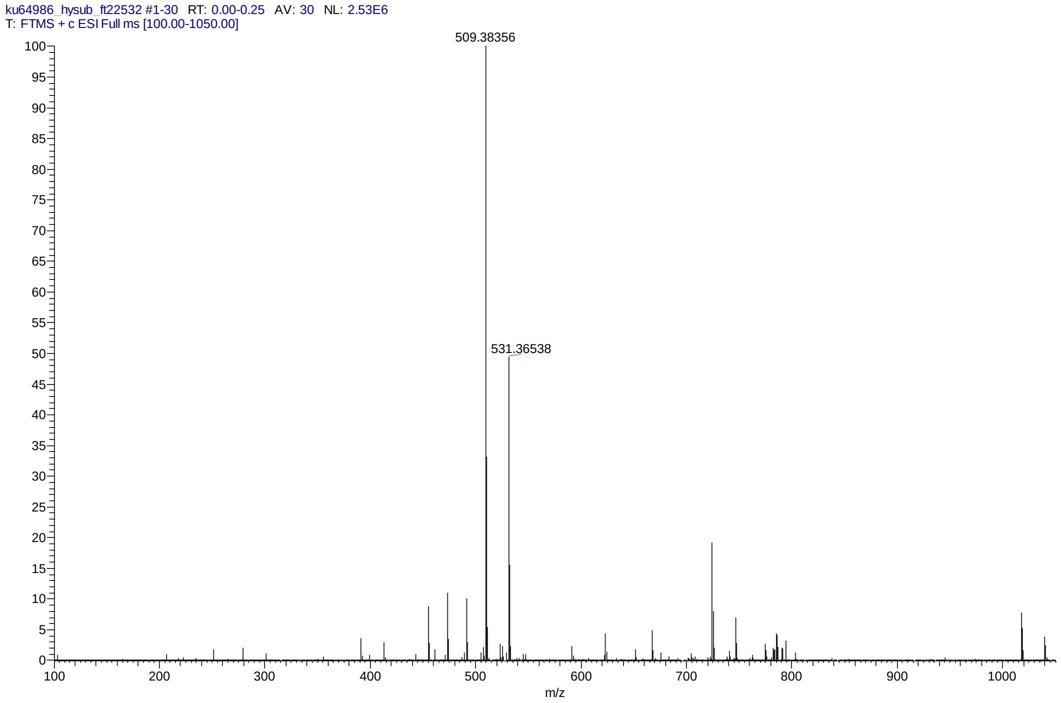

Figure S28. Full HRMS spectrum of compound **10**.

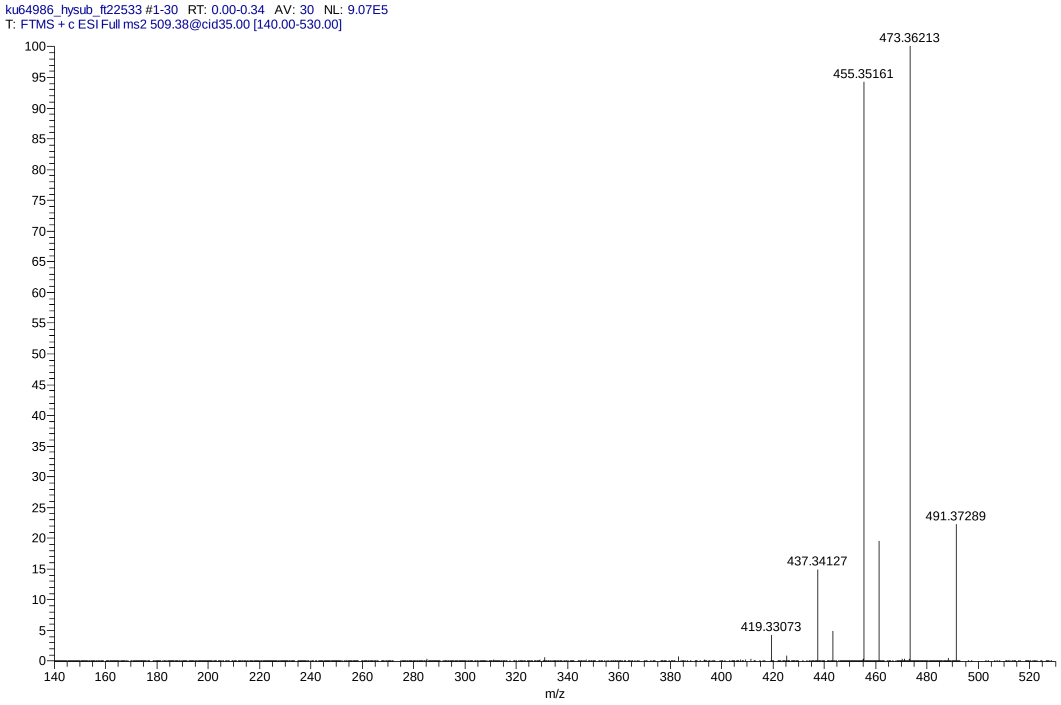

Figure S29. HR-MS/MS spectrum of compound **10**.

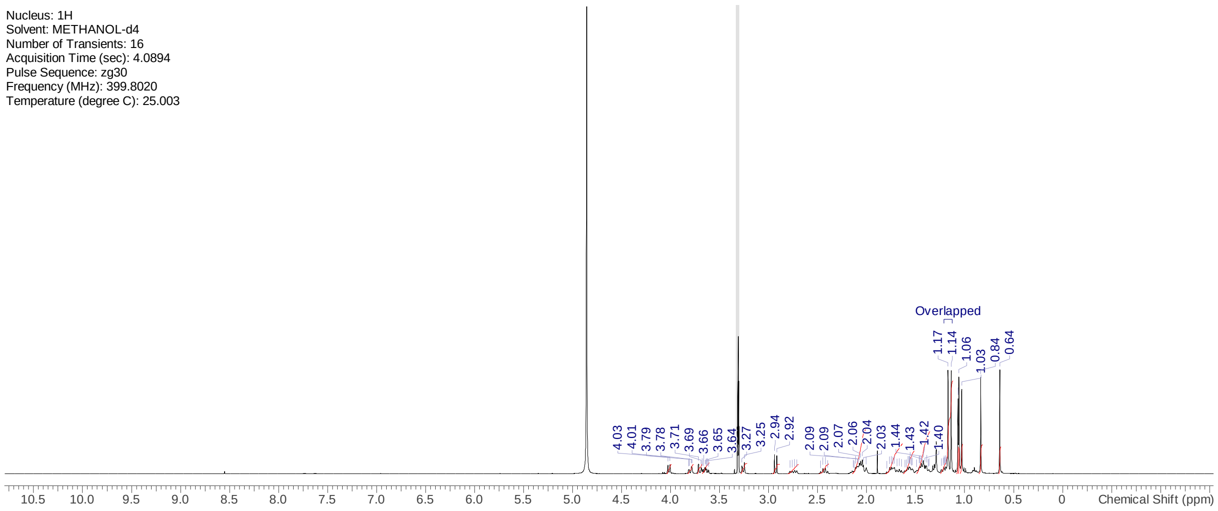

Figure S30. The ^{1}H spectrum of compound **10**.

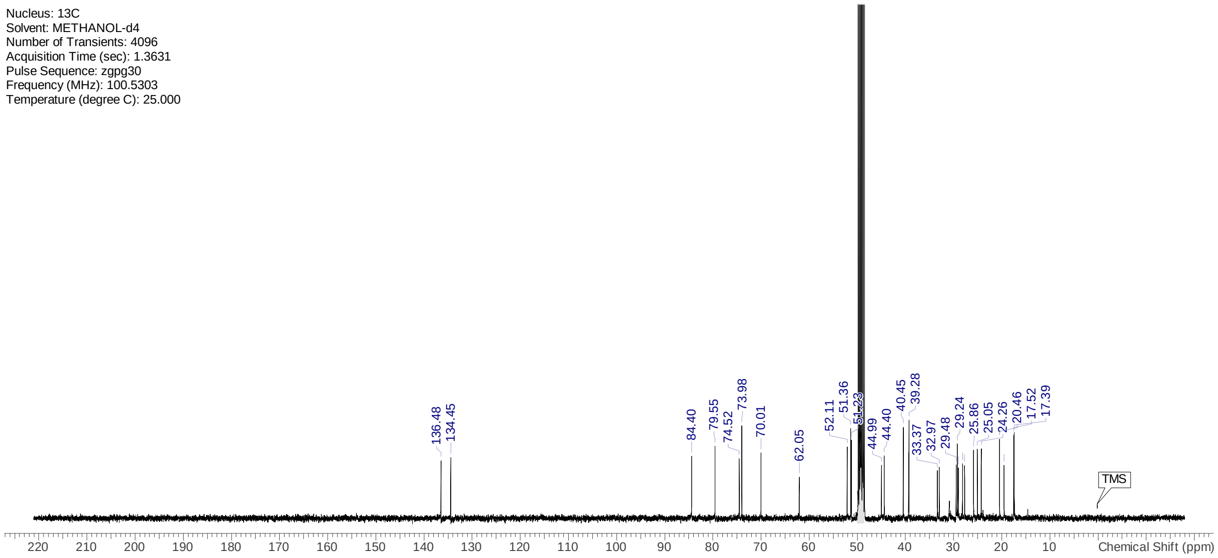

Figure S31. The ^{13}C spectrum of compound **10**.

NMR and MS Spectra and Spectral Data of Compound 11

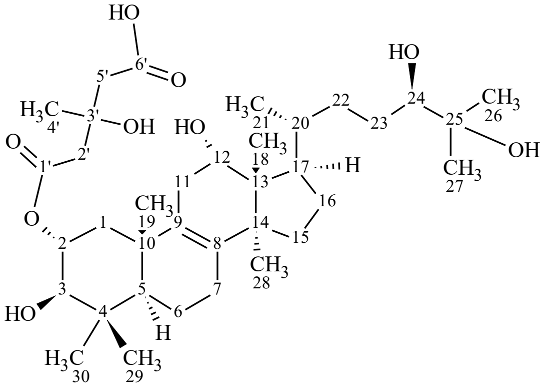

HRMS: M-H=635.41658 (delta=0.2 ppm; $C_{36}H_{59}O_9$). HR-ESI-MS-MS (CID=45%; rel. int. %): 573(68); 533(100); 515(3); 491(19); 473(4).

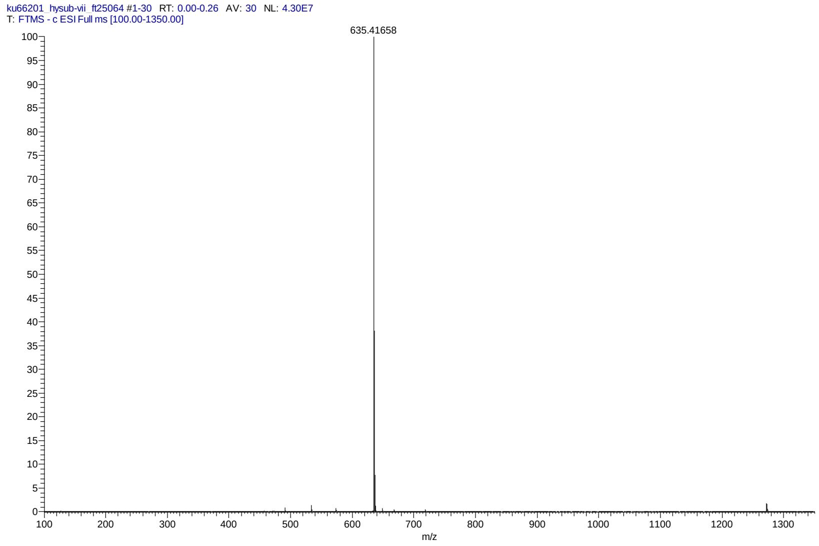

Figure S32. Full HRMS spectrum of compound **11**.

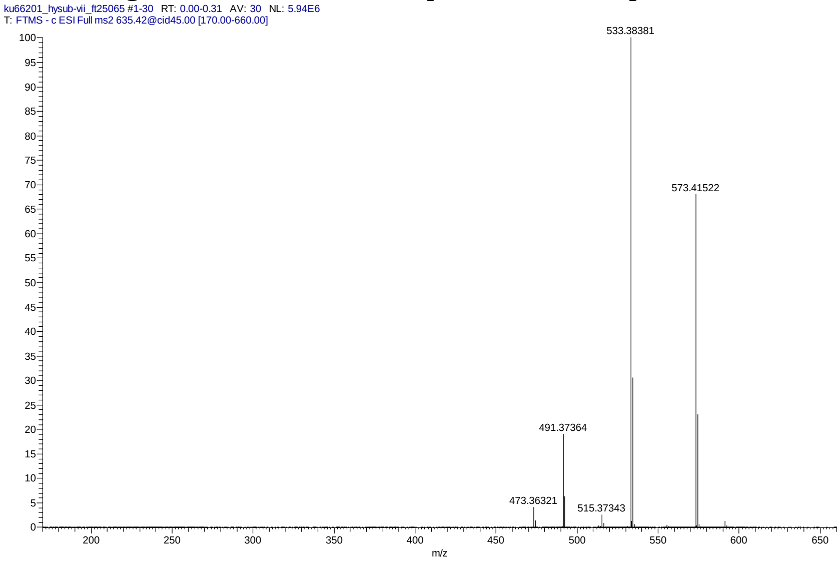

Figure S33. HR-MS/MS spectrum of compound **11**.

Table S3. NMR assignments of compound **11**.

Trivial name		Fasciculic acid B		
Atom#		C Shift	H Shift	Multiplicity (J Hz)
1	α	42.2	1.24	m
1	β	42.2	2.09	m
2		74.5	5.00	ddd (11.6, 10.2, 4.3)
3		81.0	3.20	d (10.1)
4		40.8		
5		51.9	1.21	m
6	α	19.5	1.58	m
6	β	19.5	1.74	m
7		27.6	2.10	m
8		136.9		
9		133.7		
10		39.4		
11	α	34.6	2.02	m
11	β	34.6	2.62	m
12		73.7	4.00	d (7.6)
13		50.7		
14		50.9		
15	α	33.4	1.69	m
15	β	33.4	1.17	m
16	α	29.1	2.04	m
16	β	29.1	1.38	m
17		44.2	2.21	m
18		17.1	0.66	s
19		20.4	1.11	s
20		37.7	1.44	m
21		18.1	1.03	d (6.4)
22		34.5	1.36	m
22		34.5	1.53	m
23		29.1	1.38	m
23		29.1	1.52	m
24		79.9	3.23	dd (10.0, 1.5)
25		74.1		
26		25.8	1.17	s
27		25.1	1.14	s
28		25.4	1.10	s
29		17.3	0.88	s
30		29.1	1.06	s
1'		172.8		
2'		46.9	2.71	br s
3'		71.1		
4'		28.0	1.41	s
4'		46.0	2.66	m (AB?)
5'		175.5		

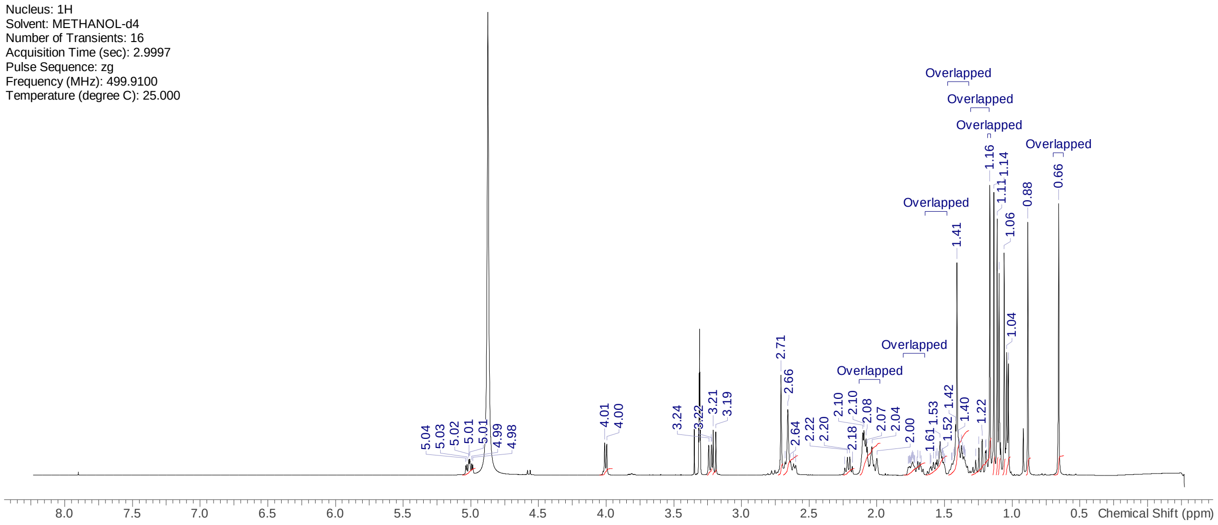

Figure S34. The ^{1}H spectrum of compound **11**.

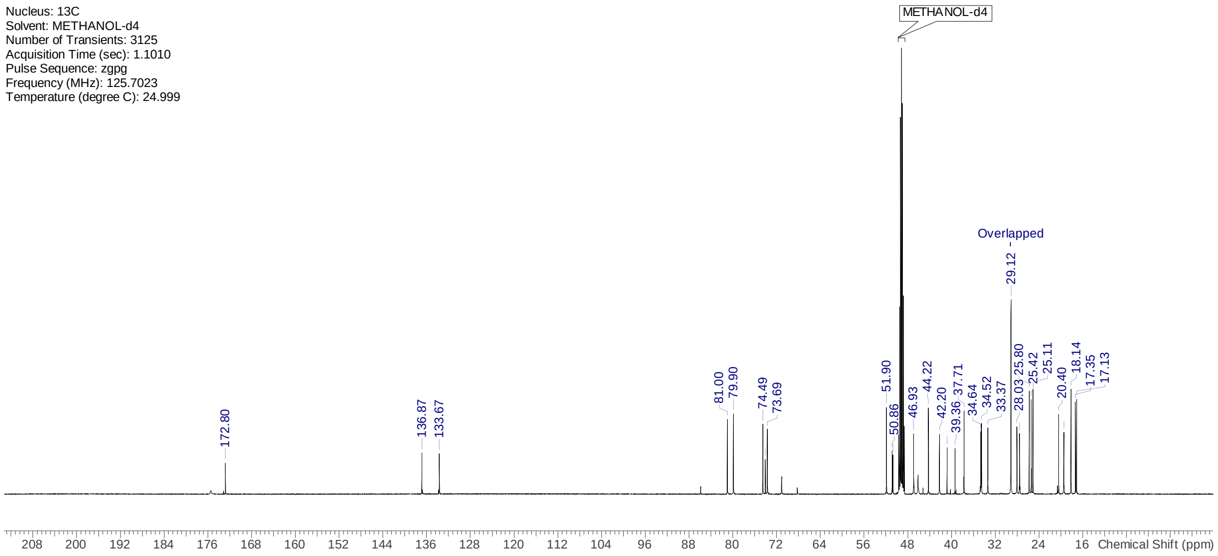

Figure S35. The ^{13}C spectrum of compound **11**.

NMR and MS Spectra and Spectral Data of Compound 12

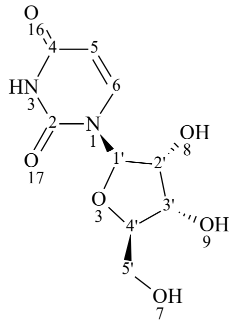

HRMS: M+Na=267.05876 (delta=0.01 ppm; $C_9H_{12}O_6N_2Na$). HR-ESI-MS-MS (CID=55%; rel. int. %): 155(20); 135(100).

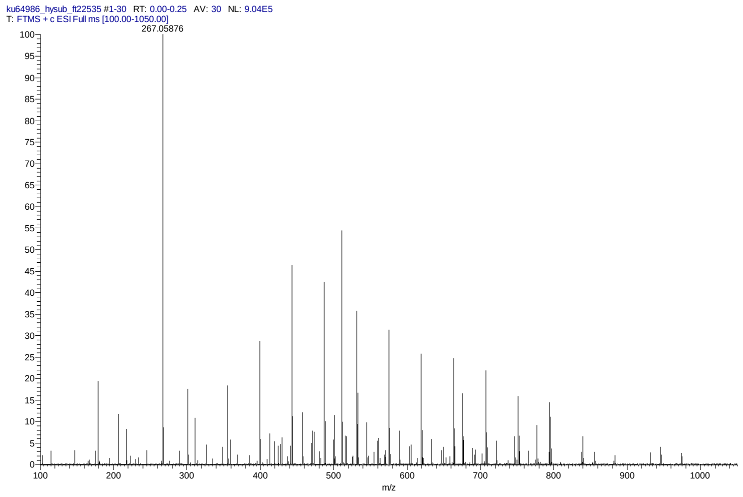

Figure S36. Full HRMS spectrum of compound **12**.

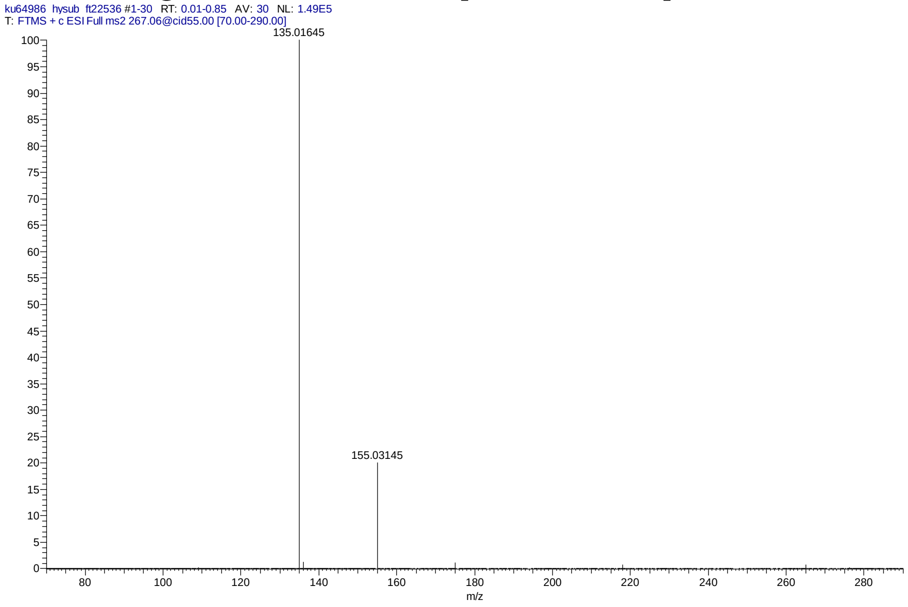

Figure S37. HR-MS/MS spectrum of compound **12**.

^{1}H NMR (400 MHz, METHANOL-d4) δ = 7.94 (1H, d, J = 8.0 Hz, H-6), 5.89 (1H, d, J = 4.4 Hz, H-1'), 5.69 (1H, d, J = 8.0 Hz, H-5), 4.14 - 4.19 (1H, m, H-2'), 4.12 - 4.16 (1H, m, H-3'), 4.00 (1H, dt, J = 4.6 Hz, J = 3.0 Hz, H-4'), 3.84 (1H, dd, J = 11.9 Hz, J = 2.7 Hz, H-5'), 3.73 (1H, dd, J = 11.9 Hz, J = 3.4 Hz, H-5')

^{13}C NMR (101 MHz, METHANOL-d_4) δ = 142.5 (C-6), 103.0 (C-5), 91.2 (C-1'), 86.4 (C-4'), 75.9 (C-2'), 71.4 (C-3'), 62.5 (C-5')

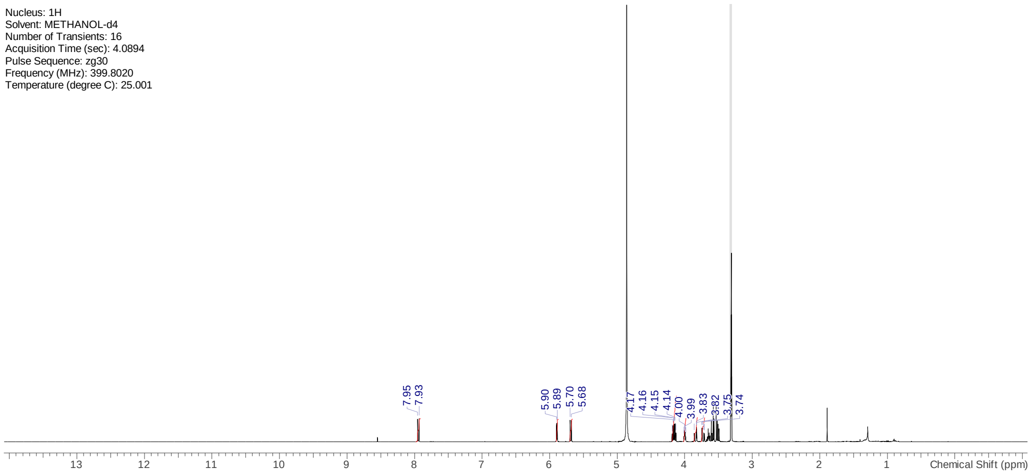

Figure S38. The ^{1}H spectrum of compound **12**.

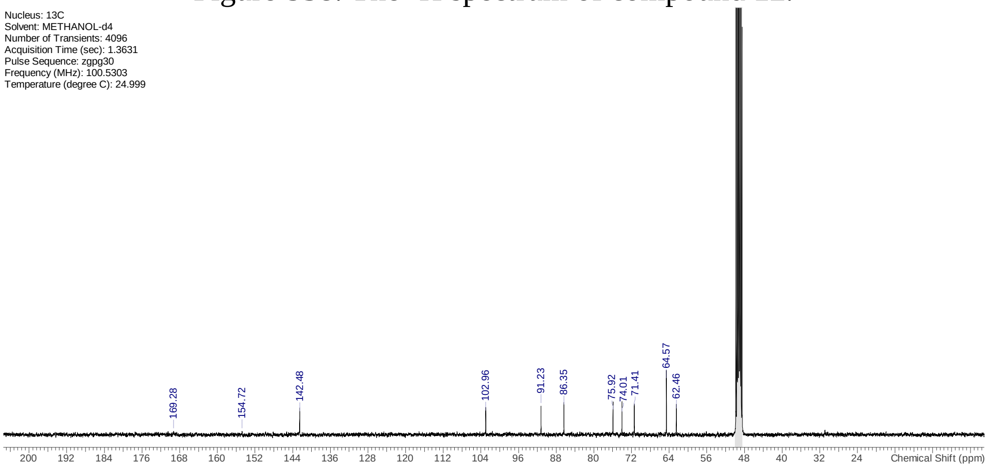

Figure S39. The ^{13}C spectrum of compound **12**.
